# Reduced sound-evoked and resting-state BOLD fMRI connectivity in tinnitus

**DOI:** 10.1016/j.nicl.2018.08.029

**Published:** 2018-08-31

**Authors:** Benedikt Hofmeier, Stephan Wolpert, Ebrahim Saad Aldamer, Moritz Walter, John Thiericke, Christoph Braun, Dennis Zelle, Lukas Rüttiger, Uwe Klose, Marlies Knipper

**Affiliations:** a Department of Otolaryngology, Head and Neck Surgery, Hearing Research Center Tübingen, Molecular Physiology of Hearing, University of Tübingen, Elfriede-Aulhorn-Str. 5, D-72076 Tübingen, Germany; b Section of Physiological Acoustics and Communication, Department of Otolaryngology, Head and Neck Surgery, University of Tübingen, Elfriede-Aulhorn-Str. 5, D-72076 Tübingen, Germany; c Department of Diagnostic and Interventional Neuroradiology, University Hospital Tübingen, Hoppe-Seyler-Str. 3, D-73076 Tübingen, Germany; d MEG Center, University Hospital Tübingen, Otfried-Müller-Str. 47, D-72076 Tübingen, Germany; e Department of Otolaryngology, Head and Neck Surgery, Hearing Research Center Tübingen, Molecular Physiology of Hearing, University of Tübingen, Elfriede-Aulhorn-Str. 5, D-72076 Tübingen, Germany/HNO Ärzte Praxis Part GmbB, Aschaffenburg, Germany

**Keywords:** Tinnitus, ABR wave, fMRI, r-fcMRI, Cortisol, ABR, auditory brainstem response, BA, Brodmann area, BA13P, posterior insula, BA13A, anterior insula, BA28, entorhinal cortex, BB-chirp, broadband chirp, BERA, brainstem-evoked response audiometry, CN, cochlear nucleus, CSF, cerebrospinal fluid, DL, dorsolateral, EFR, envelope-followed responses, ENT, ear, nose and throat, FA, flip angle, FDR, false discovery rate, FOV, field of view, FWHM, full width at half maximum, G-H-S, Goebel-Hiller-Score, HF-chirp, high-frequency chirp, High-SR AF, high-spontaneous firing rates auditory fibers, HPA, hypothalamic-pituitary-adrenal, IC, inferior colliculus, L, left, LF-chirp, low-frequency chirp, Low-SR AF, low-spontaneous firing rates auditory fibers, M, medial, MGB, medial geniculate body, MNI, Montreal Neurological Institute, PFC, prefrontal cortex, PTA, pure tone audiogram, R, right, rCBF, resting-state cerebral blood flow, rCBV, resting-state cerebral blood volume, ROI, region of interest, SD, standard deviation, SOC, superior olivary complex, SPL, sound pressure level, SPM, Statistical Parametric Mapping, TA, acquisition time, TE, echo time, TR, repetition time, VBM, voxel-based morphometry, zFC, z-values functional connectivity

## Abstract

The exact neurophysiological basis of chronic tinnitus, which affects 10-15% of the population, remains unknown and is controversial at many levels. It is an open question whether phantom sound perception results from increased central neural gain or not, a crucial question for any future therapeutic intervention strategies for tinnitus.

We performed a comprehensive study of mild hearing-impaired participants with and without tinnitus, excluding participants with co-occurrences of hyperacusis. A right-hemisphere correlation between tinnitus loudness and auditory perceptual difficulty was observed in the tinnitus group, independent of differences in hearing thresholds. This correlation was linked to reduced and delayed sound-induced suprathreshold auditory brain responses (ABR wave V) in the tinnitus group, suggesting subsided rather than exaggerated central neural responsiveness. When anatomically predefined auditory regions of interest were analysed for altered sound-evoked BOLD fMRI activity, it became evident that subcortical and cortical auditory regions and regions involved in sound detection (posterior insula, hippocampus), responded with reduced BOLD activity in the tinnitus group, emphasizing reduced, rather than increased, central neural gain. Regarding previous findings of evoked BOLD activity being linked to positive connectivities at rest, we additionally analysed r-fcMRI responses in anatomically predefined auditory regions and regions associated with sound detection. A profound reduction in positive interhemispheric connections of homologous auditory brain regions and a decline in the positive connectivities between lower auditory brainstem regions and regions involved in sound detection (hippocampus, posterior insula) were observed in the tinnitus group. The finding went hand-in-hand with the emotional (amygdala, anterior insula) and temporofrontal/stress-regulating regions (prefrontal cortex, inferior frontal gyrus) that were no longer positively connected with auditory cortex regions in the tinnitus group but were instead positively connected to lower-level auditory brainstem regions. Delayed sound processing, reduced sound-evoked BOLD fMRI activity and altered r-fcMRI in the auditory midbrain correlated in the tinnitus group and showed right hemisphere dominance as did tinnitus loudness and perceptual difficulty. The findings suggest that reduced central neural gain in the auditory stream may lead to phantom perception through a failure to energize attentional/stress-regulating networks for contextualization of auditory-specific information. Reduced auditory-specific information flow in tinnitus has until now escaped detection in humans, as low-level auditory brain regions were previously omitted from neuroimaging studies.

Trial registration: German Clinical Trials Register DRKS0006332.

## Highlights

• Tinnitus is linked with reduced rather than enhanced central sound responsiveness.
• Tinnitus allies with reduced sound-evoked BOLD fMRI activity in the auditory cortex.
• Tinnitus joins reduced connectivity between lower and higher level auditory regions.
• Tinnitus combines altered connectivities to stress-regulating frontal regions.
• Changed stress-regulating regions in tinnitus may cause blunted cortisol levels.

## 1 Introduction

Tinnitus, a phantom auditory sensation, affects approximately 10-15 % of the general population.

It is generally accepted that tinnitus is linked to increased spontaneous firing rates following deafferentation of auditory nerves (Brozoski et al., 2002; Chen and Jastreboff, 1995; Eggermont, 2012; Eggermont, 2015; Eggermont and Roberts, 2015; Kalappa et al., 2014; Kaltenbach et al., 2004; Kwon et al., 1999; Norena and Eggermont, 2003; Schaette and Kempter, 2012a; Shore et al., 2016; Weisz et al., 2005; Weisz et al., 2007; Yang and Bao, 2013). However, the tinnitus literature features various proposed neural correlates of how elevated spontaneous activity is connected to tinnitus. Almost all of the literature currently assumes that the generation of the elevated spontaneous activity is correlated with the percept of tinnitus through increased central neural gain in lower or higher brain levels (Marks et al., 2018; Noreña, 2015; Schaette and Kempter, 2012a; Schaette and McAlpine, 2011a; Sedley et al., 2016; Yang and Bao, 2013; Yang et al., 2011). Within this view deafferented regions may generate increases in the discharge rate in the brainstem to compensate for deprived auditory input (Noreña, 2015; Schaette and Kempter, 2012a; Schaette and McAlpine, 2011a). This is suggested to lead to elevated cortical activity essential for perception of tinnitus following disinhibition along the auditory path and auditory cortex (Roberts et al., 2010) accompanied by increased correlations with the SFR (Eggermont and Roberts, 2012).

Other studies, although not yet widely or independently supported, have instead suggested that the elevated spontaneous activity in tinnitus rather leads to failure to increase central neural gain, which is associated with a reduced signal-to-noise ratio and elevated noise levels (Knipper et al., 2013; Rüttiger et al., 2013a; Singer et al., 2013a; Zeng, 2013). A failure to increase central neural gain is hypothesized to be related to a critical loss of high-SR, low-threshold fibers, based on a high degree of IHC ribbon loss in animals with behavioral tinnitus (Rüttiger et al., 2013b; Singer et al., 2013b) and based on a mice mutant with a loss of specific auditory fiber characteristics and reduced tonic inhibitory strength in the ascending pathways (Chumak et al., 2016). As a consequence of the more severe damage to the IHC synapse in animals with tinnitus, the amplitudes of central ABR waves do not restore and molecular markers for plasticity of synaptic strength are not mobilized (Rüttiger et al., 2013b; Singer et al., 2013b), a feature that may be linked to elevated noise levels through reduced tonic inhibitory strength (Chumak et al., 2016).

This question is fundamental for a therapeutic intervention strategy for tinnitus that aims to influence the central imbalances in excitability present within the auditory pathway.

To shed more light on this existing inconsistency, we performed a multimodal dataset analysis of participants with tinnitus and volunteers without tinnitus. Aiming to maximize information about auditory-specific changes in tinnitus, the study design aligned with several *a priori* assumptions (i) we focused on mild hearing-impaired volunteers and participants with tinnitus with hearing thresholds <40 dB in order to obtain homogenous groups (Knipper et al., 2013; Shore et al., 2016); (ii) we excluded participants with co-occurrences of tinnitus and hyperacusis which may disturb interference through dissimilar central neural responses (Gu et al., 2010; Song et al., 2014); (iii) as hearing-impaired matched rats with and without tinnitus have been shown to differ in terms of the size of suprathreshold central auditory brainstem response (ABR) waves independent of hearing thresholds (Rüttiger et al., 2013a), we included detection of suprathreshold ABR waves; (iv) As the sound-induced (ABR) wave size (wave amplitude) reflects synchronized neural activity (Johnson and Kiang, 1976; Ruttiger et al., 2017), we included BOLD fMRI activity, which is known to change in response to a task requiring elevated local metabolism (Logothetis et al., 2001); (v) as an increased level of evoked BOLD fMRI activity has been previously linked to more synchronous fMRI correlations at rest (Haag et al., 2015), we hoped to strengthen the obtained findings through additional analyses of resting-state functional connectivity MRI (r-fcMRI) in anatomically predefined auditory pathway and connected regions; and (vi) finally, the accepted influence of corticosterone levels on early and late ABR waves after tinnitus-inducing trauma (Singer et al., 2018; Singer et al., 2013a) and the positive association between glucocorticoid resistance and tinnitus (Hébert et al., 2012; Mazurek et al., 2012), motivated us to analyze the cortisol levels of each participant.

Alternatively, regarding higher-level central neural gain as a neural correlate for tinnitus generation, our findings rather support reduced auditory response gain as a neural correlate of tinnitus. This response change has previously escaped attention in tinnitus patients, as lower auditory brainstem regions were not routinely imaged. The findings provide candidate neural correlates for predicted tinnitus precursors in previous tinnitus models (Jastreboff, 1999b; Sedley et al., 2016) that are discussed in the context of current tinnitus therapies.

## 2 Materials and methods

### 2.1 Participants

From 58 participants 34 were included in the study based on hearing thresholds not >40 dB per single frequency in the pure tone audiogram (PTA) and hyperacusis questionnaire outcome (see including and exclusion criteria Supplementary Table S2).

### 2.2 Tinnitus questionnaire

The Goebel-Hiller-Score (G-H-S) tinnitus questionnaire was used to assess different aspects concerning tinnitus severity, laterality, emotional distress, cognitive distress, self-experienced intrusiveness, and auditory perceptual difficulty scores (Hiller et al., 1994) as described under methods (see for detail Supplementary material). In order to assess the presence of hyperacusis, a Hyperacusis Questionnaire (Fischer, 2013) was administered to all participants.

### 2.3 Audiological evaluation

Ear examination, tympanometry, acoustic reflex measurements, pure tone audiometry and speech audiometry were determined as described in Supplementary material. The auditory evoked brainstem response (ABR) testing was done by using a brainstem evoked response audiometry (BERA) system as described in detail in Supplementary material.

### 2.4 Calculation of supra-threshold ABR wave fine structure

From the averaged ABR of a single ear and stimulus SPL, the absolute latencies and amplitudes of distinct components within the waveform of the ABR were extracted and attributed as described in detail in Supplementary material.

### 2.5 Salivary cortisol measurement

Salivary cortisol levels were measured as the most reliable body fluid for detecting a hypothalamic-pituitary-adrenal (HPA) axis imbalance (Elder et al., 2017; Verbeeten and Ahmet, 2017). The salivary cortisol level was measured at three different day times (08 a.m., 04 p.m. and 11 p.m.) as described in Supplementary material.

### 2.6 Functional magnetic resonance imaging (fMRI)

fMRI image acquisition was performed on a 3-Tesla scanner (Skyra, Siemens, Germany). For the acoustic stimulation, we used special MRI-suitable over-ear headphones (CONFON HP-SC 03, MR Confon GmbH, Magdeburg, Germany). During scanning, five different auditory stimuli were generated using a stimulus presentation software (Neurobehavioral Systems software, Neurobs, Berkeley, USA; Panasonic-SC-PMX5 Amplifier, Panasonic Marketing Europe GmbH, Hamburg, Germany).

### 2.7 The measurement experimental design of the task fMRI

The task-evoked functional images were obtained with a T2* weighted echo-planar sequence. Cardiac gating was used to avoid signal distortion due to brain pulsation induced by the heart function especially near the brainstem. Here, we used a specific repetition time (TR)-correction procedure, aiming to maximally reduce variable TRs that would obscure the BOLD signal, e.g., in lower-level auditory brainstem regions via vascular pulsations during cardiac gating (Guimaraes et al., 1998). fMRI BOLD activity was calculated in regions that were defined prior to the fMRI using anatomical datasets. Exact measurement parameters are described in detail under Supplementary material (Task-evoked fMRI). Aiming at measuring the BOLD, in response to different acoustic stimuli, the presenter software version 16.1 (Neurobehavioral Systems software) was used to present four different acoustic stimuli with a specially written protocol. The stimuli used are (1) high-frequency chirp (HF-chirp), (2) low-frequency chirp (LF-chirp), (3) broadband chirp (BB-chirp) and (4) a rock music piece. The pulse oximeter was applied to one of the participant's fingers to synchronize the heartbeats with the image acquisition. Task (evoked) functional magnetic resonance imaging analysis was performed as described in detail under Supplementary material.

### 2.8 The measurement experimental design of the resting-state fMRI

The resting-state functional images for the whole brain were acquired over a 10 min’ acquisition time (TA) period of awake rest. Measurement parameters beside repetition time (TR) (2 s) and number of slices (30) were identical to task evoked. To get particular information about the resting-state differences in lower brainstem (cochlear nucleus (CN)), superior olivary complex (SOC), inferior colliculus (IC), the field of view (FOV) block (normally aligned to anterior commissure (AC) – posterior commissure (PC) line) of the excited slices were moved toward lower brain regions. This technical adaptation is novel in the investigation of the auditory system using r-fcMRI studies.

The participants were instructed to remain alert with their eyes closed, with no task to perform. Earplugs were used for all participants during the scan to reduce noise generated by the scanner. 300 volumes were acquired. Exact measurement parameters as well as r-fcMRI analysis was performed as described in detail in Supplementary material.

### 2.9 Statistical analysis

Unless otherwise noted, statistical significance was tested at the level of α = 5%. Level of significance is illustrated in the figures with symbols or shaded areas (not significant (n.s.): *p* *>* *0.05*; *: *p* *<* *0.05*; **: *p* *<* *0.01*; ***: *p* *<* *0.001*). For all statistical tests, MATLAB was used for evaluation. All fMRI results are False Discovery Rate (FDR) of *p* *<* *0.05* corrected.

**Fig. 1:**
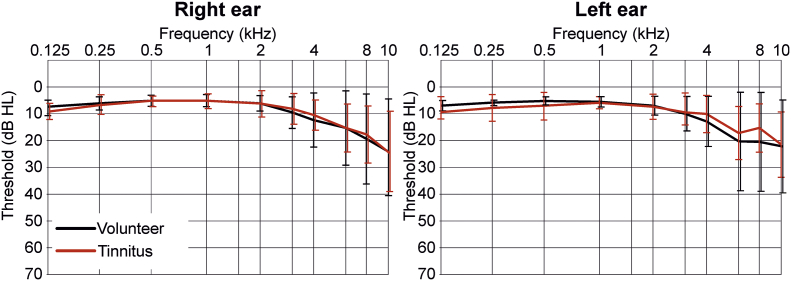
Pure tone audiometry. Averaged pure tone audiometry (mean ± SD) of all volunteer (black) and tinnitus (red) participants of right and left ears separately. Three-way ANOVA with repetition was used, *n* *=* *17* for each group (volunteer and tinnitus). There is no statistical difference between the volunteer and tinnitus group in hearing threshold level at any frequency. Frequency *F* *=* *30.7*, *p* *=* *0.0001*, group *F* *=* *0.34*, *p* *=* *0.56*; ear side *F* *=* *0.18*, *p* *=* *0.67*; frequency*group *F* *=* *0.54*, *p* *=* *0.84*; frequency*ear side *F* *=* *0.35*, *p* *=* *0.95*; frequency*group*ear side *F* *=* *0.14*, *p* *=* *0.99*.

For pure tone audiometry (Fig. 1) data are presented as mean with SD for *n* *=* *17* volunteers and *n* *=* *17* tinnitus (for age, sex, handedness see Supplementary Table S1A). One-sample Kolmogorov-Smirnov test was used to test each group for normal distribution. Three-way ANOVA with repetition was used to test for differences in hearing threshold between both groups in frequencies from (0.125 kHz to 10 kHz). ANOVA test was followed by Tukey's multiple comparisons test with the α-level of 0.05.

**Fig. 2:**
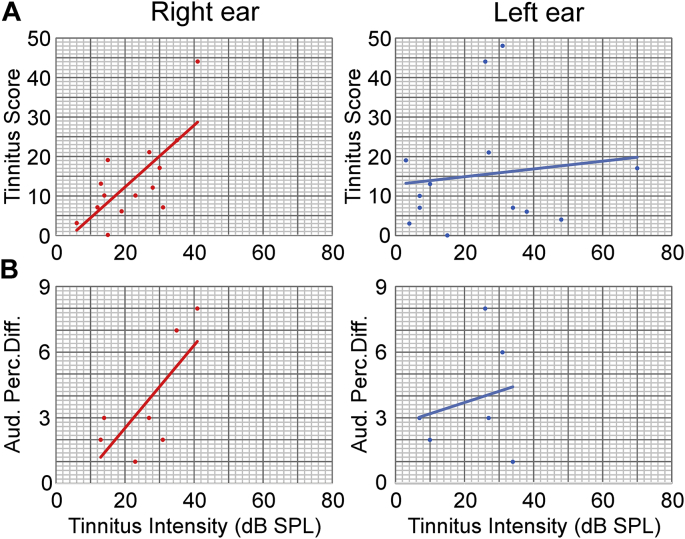
Tinnitus-Questionnaire correlation. (A) Correlation of tinnitus loudness (*n* depends on the perception of tinnitus in the respective ear) with total Tinnitus-Questionnaire score for right and left ears. *n* *=* *14* for the right ear, *n* *=* *13* for the left ear. The tinnitus intensity is positively correlated with the total tinnitus score for right ear (*R* *=* *0.68; p* *<* *0.01*) but not for the left ear (*R* *=* *0.11; p* *=* *0.05*). (B) Correlation of tinnitus loudness (*n* depends on the perception of tinnitus in the respective ear) with auditory perceptual difficulty questionnaire score for right and left ears. *n* *=* *7* for the right ear, *n* *=* *6* for the left ear. The tinnitus intensity is positively correlated with the auditory perceptual difficulty score for right ear (*R* *=* *0.79; p* *<* *0.05*) but not for the left ear (*R* *=* *0.25; p* *>* *0.05*).

For Tinnitus-Questioner correlation (Fig. 2) data are presented as single value – *n* depends on the perception of tinnitus, Pearson Correlation was used. Fig. 2A shows total tinnitus score over perceived tinnitus intensity. For the right ear *n* *=* *14* (for age, sex, handedness see Supplementary Table S1A). For the left ear *n* *=* *13* (for age, sex, handedness see Supplementary Table S1A). Fig. 2B shows auditory perceptual difficulty over perceived tinnitus intensity. For the right *n* *=* *7* (for age, sex, handedness see Supplementary Table S1A). For the left ear *n* *=* *6* (for age, sex, handedness see Supplementary Table S1 A).

**Fig. 3:**
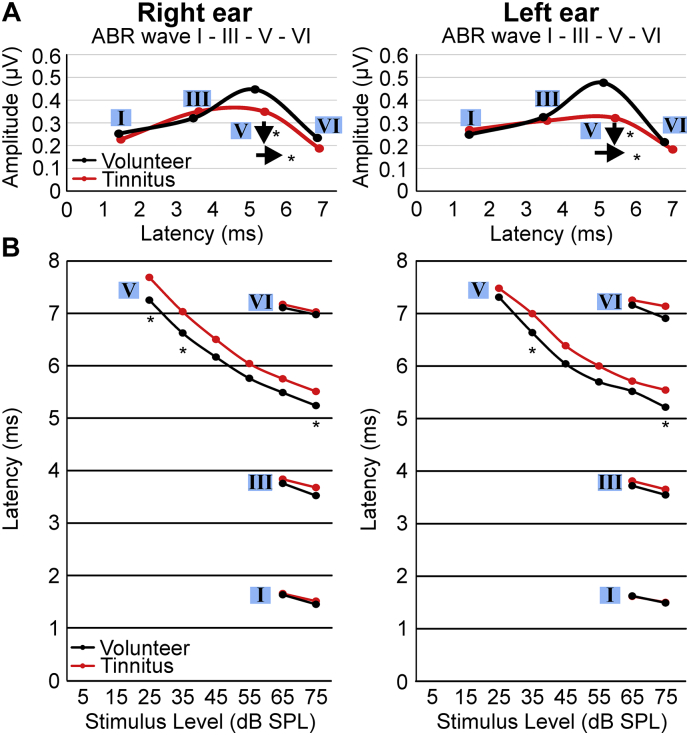
Amplitude reduction in ABR wave/Latency shift in ABR wave. (A) Averaged ABR wave I, III, V, and VI latency-supra-threshold amplitude plot at 75 dB SPL for volunteer and tinnitus group of right and left ears separately. Amplitudes of the waves are connected via spline. Three-way ANOVA with repetition was used. Each group *n* *=* *17*. Tukey's multiple comparison tests – for wave V significant amplitude reduction in tinnitus for the right ear (volunteer 0.45 ± 0.16 μV; tinnitus 0.34 ± 0.09 μV; *p* *=* *0.046*) and for the left ear (0.48 ± 0.17 μV; tinnitus 0.32 ± 0.08 μV; *p* *=* *0.02*). (B) Intensity-Latency function curve for averaged group ABR waves I, III, V, and VI (at 75 and 65 dB SPL; Wave V also at 55, 45, 35 and 25 dB SPL) of the right and left ears separately. Three-way ANOVA with repetition was used. Each group *n* *=* *17*. Tukey's multiple comparison tests – for wave V significant latency shift in tinnitus for right ear (volunteer 5.23 ± 0.45 ms; tinnitus 5.50 ± 0.23 ms; *p* *=* *0.034*) and for the left ear (5.22 ± 0.26 ms; tinnitus 5.54 ± 0.20 ms; *p* *=* *0.016*) at 75 dB SPL. For the right ear at 35 (*p* *=* *0.02*), 25 (*p* *=* *0.013)*, for the left ear at 35 (*p* *=* *0.02*) significant latency shift between tinnitus and volunteer. ABR, auditory brainstem response; dB, decibel; SPL, sound pressure level; μV, microvolt; ms, milliseconds.

For ABR wave measurement (Fig. 3A) data are presented as mean amplitude values for wave I, III, V and VI over latency (lines between these points are approximated for visualization) for *n* *=* *17* volunteers and *n* *=* *17* tinnitus (for age, sex, handedness see Supplementary Table S1A). Due to the measured ABR signal, it was only for wave V feasible to get ABR responses far low the supra-threshold SPL down to 25 dB SPL. For wave I, III and VI, only SPL ABR responses at 75 and 65 dB were recorded. One-sample Kolmogorov-Smirnov test was used to test the normal distribution for amplitude and latency in each wave for both groups. Three-way ANOVA with repetition was used to test the wave amplitude and latency for significant difference between both groups, in relation to the ear-side. ANOVA test was followed by Tukey's multiple comparisons test with the α-level of 0.05.

For ABR Latency (Fig. 3B) data are presented as mean latency values for wave I, III, V and VI over the applied SPL intensity (lines between these points are approximated for visualization) for *n* *=* *17* volunteers and *n* *=* *17* tinnitus (for age, sex, handedness see Supplementary Table S1A). One-sample Kolmogorov-Smirnov test was used to test the normal distribution for latency in each wave over the intensity for both groups. Three-way ANOVA with repetition was used to test the mean latency value for significant difference between both groups. ANOVA test was followed by Tukey's multiple comparisons test with the α-level of 0.05.

**Fig. 4:**
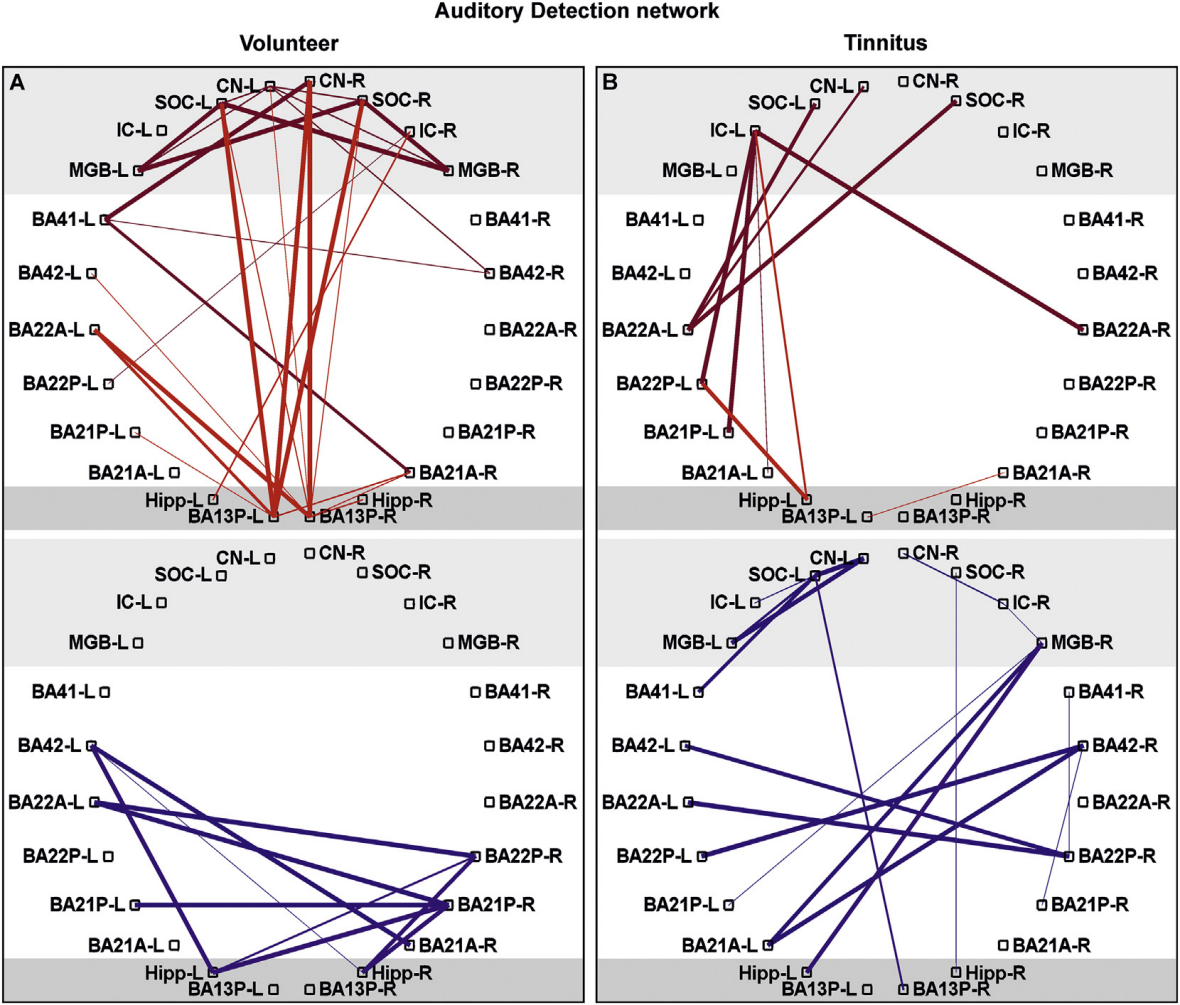
Functional connectivity pattern of defined auditory brain regions of interest. Significant Pearson correlation of BOLD dependent time courses of different brain regions shown for volunteer (A) and tinnitus (B) group (*n* *=* *17*; *p* *<* *0.05*) for positive (upper panel red) and negative (lower panel blue) connectivity. The darker red/blue is for connectivities between lower and higher level auditory nuclei in right and left hemispheres and brighter red/blue for connectivities between regions participating to sound detection as the posterior insula (BA13P) and the hippocampus. The thickness of lines corresponds to the correlation-value. R, right; L, left; CN, cochlear nucleus; SOC, superior olivary complex; IC, inferior colliculus; MGB, medial geniculate body; BA, Brodmann area; A, anterior; P, posterior; Hipp, hippocampus; BOLD, blood oxygenation level depended.

**Fig. 5:**
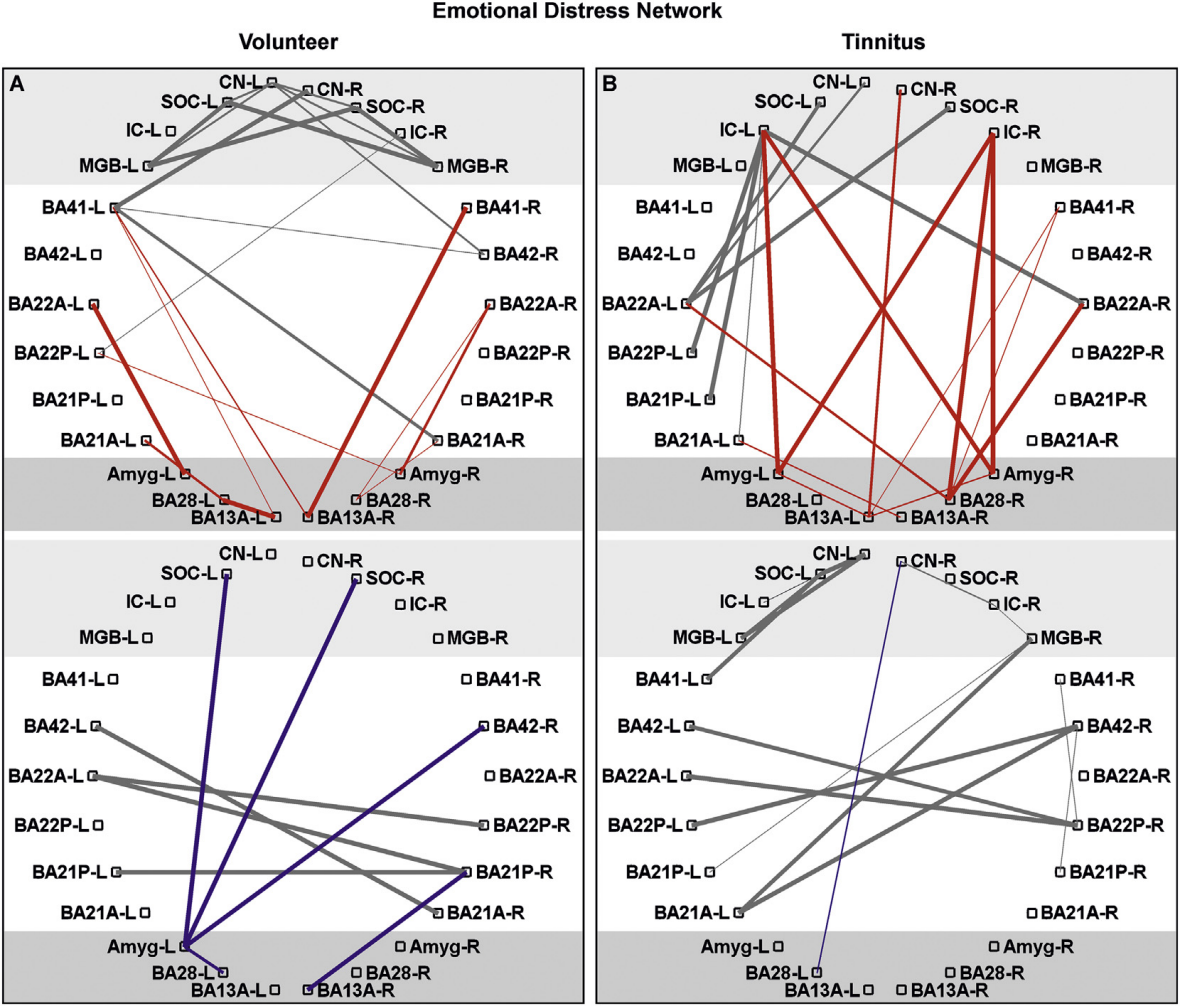
Functional connectivity pattern of defined auditory and limbic/distress brain regions of interest. Significant Pearson correlation of BOLD dependent time courses of different brain regions shown for volunteer (A) and tinnitus (B) group (*n* *=* *17*; *p* *<* *0.05*) for positive (upper panel red) and negative (lower panel blue) connectivity. The red/blue labels connectivities between regions of the limbic and distress system as the amygdala, entorhinal cortex (BA28) and anterior insula (BA13A). The thickness of lines corresponds to the correlation-value. The gray lines shows the connections between auditory regions from Fig. 4. R, right; L, left; CN, cochlear nucleus; SOC, superior olivary complex; IC, inferior colliculus; MGB, medial geniculate body; BA, Brodmann area; A, anterior; P, posterior; Amyg, amygdale.

**Fig. 6:**
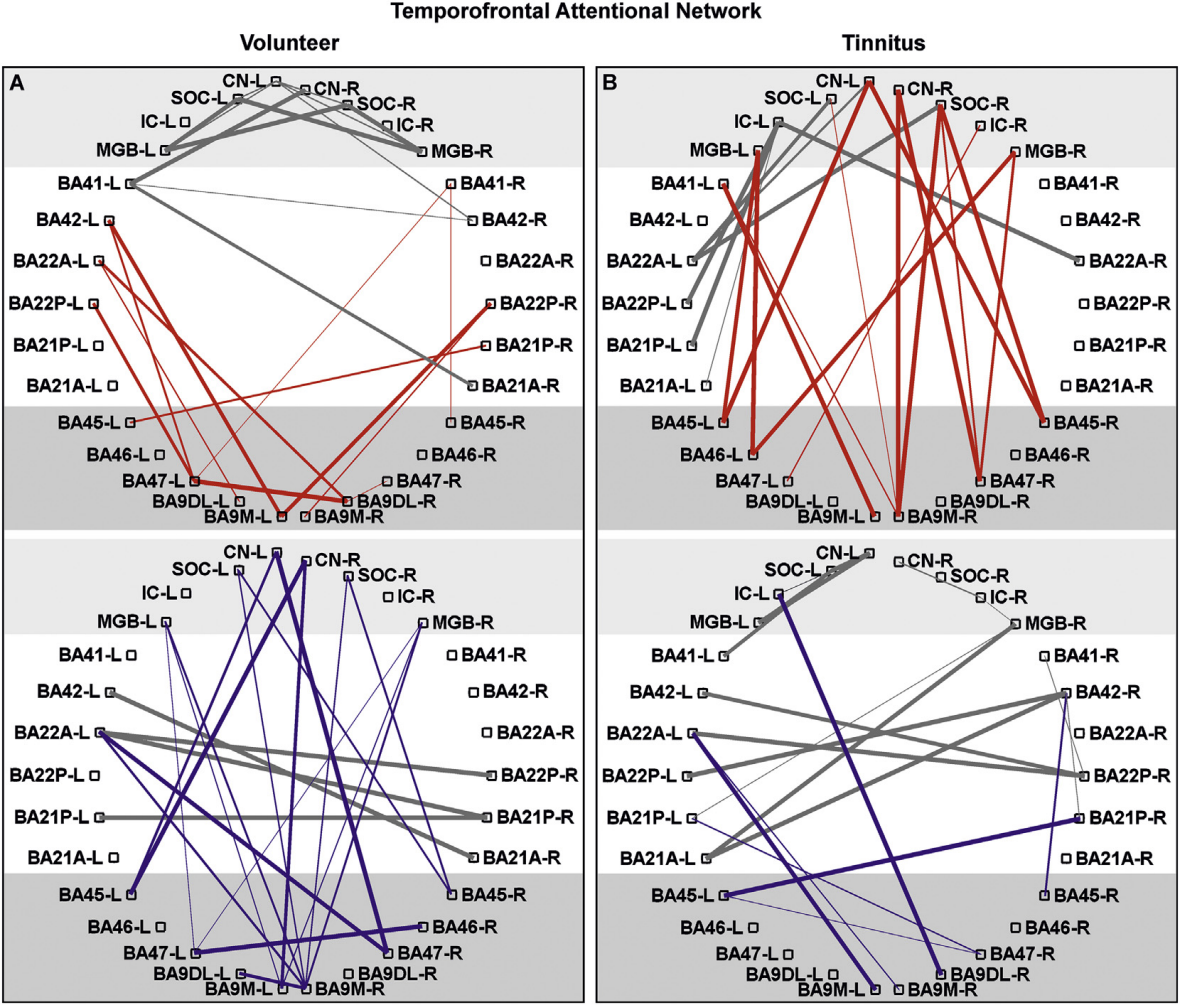
Functional connectivity pattern of defined auditory and attentional brain regions of interest. Significant Pearson correlation of BOLD dependent time courses of different brain regions shown for volunteer (A) and tinnitus (B) group (*n* *=* *17*; *p* *<* *0.05*) for positive (upper panel red) and negative (lower panel blue) connectivity. The red/blue labels connectivities between regions of the temporofrontal network, as the BA45, BA46, BA47, dorsolateral prefrontal cortex (BA9DL) and the medial prefrontal cortex (BA9M). The thickness of lines corresponds to the correlation-value. The gray lines show the connections between auditory regions from Fig. 4. R, right; L, left; CN, cochlear nucleus; SOC, superior olivary complex; IC, inferior colliculus; MGB, medial geniculate body; BA, Brodmann area; A, anterior; P, posterior; M, medial; DL, dorsolateral.

For auditory detection network (Fig. 4), emotional distress network (Fig. 5) and temporofrontal attentional network (Fig. 6) data are presented as significant *(p* *<* *0.05*, FDR corrected for defined region pairs) connectivities between region of interest (ROIs) for *n* *=* *17* volunteers and *n* *=* *17* tinnitus (for age, sex, handedness see Supplementary Table S1A).

The ROIs were extracted from a correlation matrix; therefore, the Pearson correlation was used to calculate the coefficients for the chosen ROIs in every group. The positive and negative correlation coefficients were presented separately. The averaged BOLD activity in the ROI was defined by the Montreal Neurological Institute (MNI)-coordinate with a spherical shape of 3 mm radius.

**Fig. 7:**
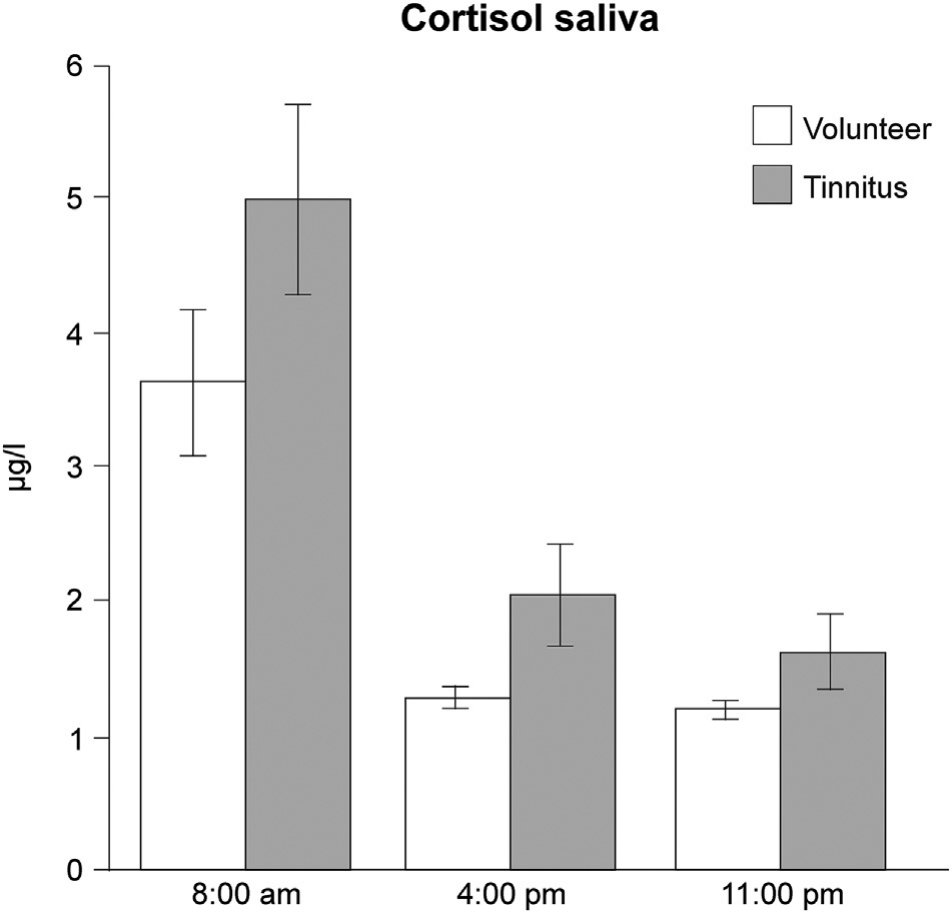
Cortisol level in saliva. The figure shows the cortisol level of saliva for *n* *=* *17*. Saliva was taken three times over the day at 8:00 a.m., 4:00 p.m. and 11:00 p.m. Two-way ANOVA with repetition was not statistically significant between volunteer and tinnitus group over the single time points. Standard error is given for every value.

For salivary measurements (Fig. 7) data are presented as mean with SD for *n* *=* *17* volunteers and *n* *=* *17* tinnitus (for age, sex, handedness see Supplementary Table S1A) at three different time points (8:00 a.m., 4:00 p.m. and 11:00 p.m.). At each time point comparisons took place between tinnitus group and volunteer group. One-sample Kolmogorov-Smirnov test was used to test each group for normal distribution. Two-way ANOVA with repetition was used for inter-group comparison over each time point. ANOVA test was followed by Sidak's multiple comparisons test with the α-level of 0.05.

For ROIs with reduced or enhanced evoked and resting-state fMRI (Table 2, Supplementary Table S3) data are presented as significant (*p* *<* *0.05*, FDR corrected) difference in defined brain region activity in group comparison, for *n* *=* *17* volunteers and *n* *=* *17* tinnitus (for age, sex, handedness see Supplementary Table S1A). The second level specification independent two-sample *t*-test of the Statistical Parametric Mapping (SPM) toolbox was used to perform the group analysis. Two different significance test procedures were used in the analysis. First, brain regions with a mean *decreased* evoked response in the tinnitus group were tested for a significant difference compared to the signal in the volunteer group. Second, brain regions showing a mean *increased* evoked response in the tinnitus group were tested for significant differences compared to the signal in the volunteer group.

For connectivities between defined ROIs in resting-state fMRI (Supplementary Table S4) data are presented as significant (*p* *<* *0.05*, FDR corrected) positive or negative correlation coefficients, for *n* *=* *17* volunteers and *n* *=* *17* tinnitus (for age, sex, handedness see Supplementary Table S1A). Data processing steps are described above. After testing against random networks (for details see Supplementary material), the Pearson correlation matrix was used to demonstrate all the significant correlations in between time courses of defined ROIs.

For fMRI power-calculation, the fMRI Power toolbox (Mumford, 2012) was used to calculate the statistical power of the fMRI study. Type I error was 0.05, group design matrix was two sample *t*-test with *n* *=* *17* for each group, ROIs were defined in an own ROI-mask. Statistical power for the defined ROIs is between 82.7 % and 86.97 %.

## 3 Results

### 3.1 Reduced late sound-induced auditory brainstem responsiveness in tinnitus

**Supplementary Fig. S1:**
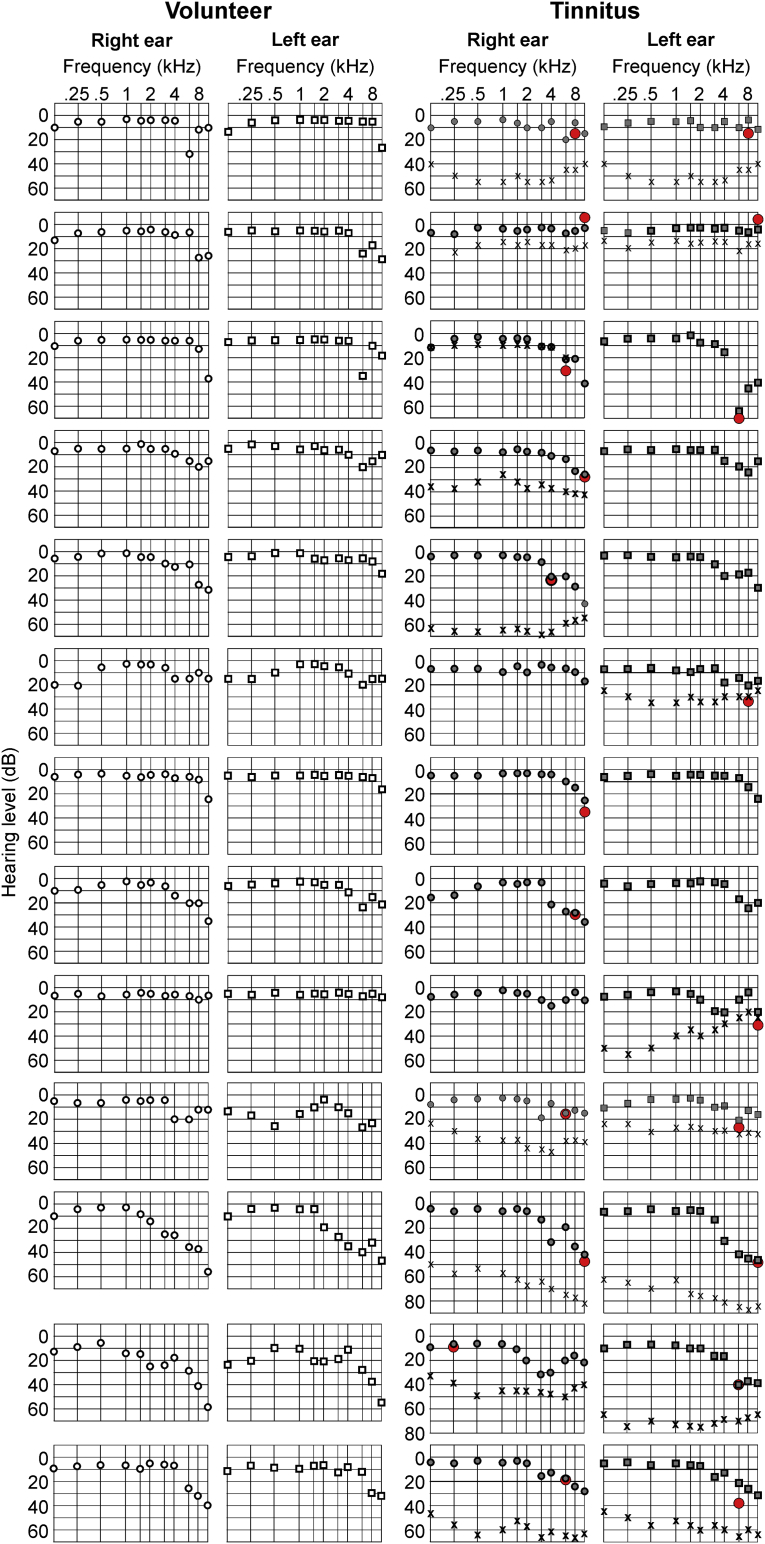
Individual pure tone audiogram. The figure shows as an example (n = 13 for each group) pure tone audiogram for participants without (left side – right and left ear) and participants with tinnitus (right side – right and left ear) from 0.125 kHz up to 10 kHz. For the tinnitus group the pure tone audiogram (right side) has additionally the tinnitus localization added (red dot) as well as the tinnitus suppression, if it was applicable (single crosses during the audiogram).

Participants with and without tinnitus (volunteers) were recruited as described in the Method section (Supplementary Table S1). Of the 29 initial participants in each group, only 17 volunteers and 17 participants with tinnitus were enrolled. Twelve participants were excluded from the tinnitus group (and 12 from the matched volunteer group) due to the co-occurrence of hyperacusis (Supplementary Table S2). The tinnitus frequency, identified as the tinnitus-masking suppression level (Supplementary material), revealed a high prevalence of tinnitus perception among all participants with tinnitus at >6 kHz. The tinnitus percept occurred with a prevalence of bilateral (65%), right- (23%), and left-side (12%) tinnitus perception (Supplementary Fig. S1). When the ABR threshold differences in pure tone audiogram (PTA) measurements between the right and left ears in the volunteers and participants with tinnitus, up to and including >10 kHz, were determined, no significant differences were seen between the hearing thresholds of the volunteers and those of the participants with tinnitus (Fig. 1 – three-way ANOVA: frequency *F* *=* *30.7*, *p* *=* *0.0001*, group *F* *=* *0.34*, *p* *=* *0.56*; ear side *F* *=* *0.18*, *p* *=* *0.67*; frequency*group *F* *=* *0.54*, *p* *=* *0.84*; frequency*ear side *F* *=* *0.35*, *p* *=* *0.95*; frequency*group*ear side *F* *=* *0.14*, *p* *=* *0.99*). As expected, frequency has a significant effect on hearing thresholds. Higher thresholds were found for frequencies >8 kHz (by Tukey's multiple comparison tests).

The outcome of the tinnitus questionnaire (G-H-S), an assessment of subjective tinnitus distress (Hiller et al., 1994) identified that neither tinnitus laterality nor tinnitus intensity were correlated with the left- or right-handedness of the participants with tinnitus. In addition, >90% of the participants (16 of 17) had low-grade (I-II) tinnitus scores (Biesinger et al., 1998b) (Supplementary Table S1A).

**Supplementary Fig. S2:**
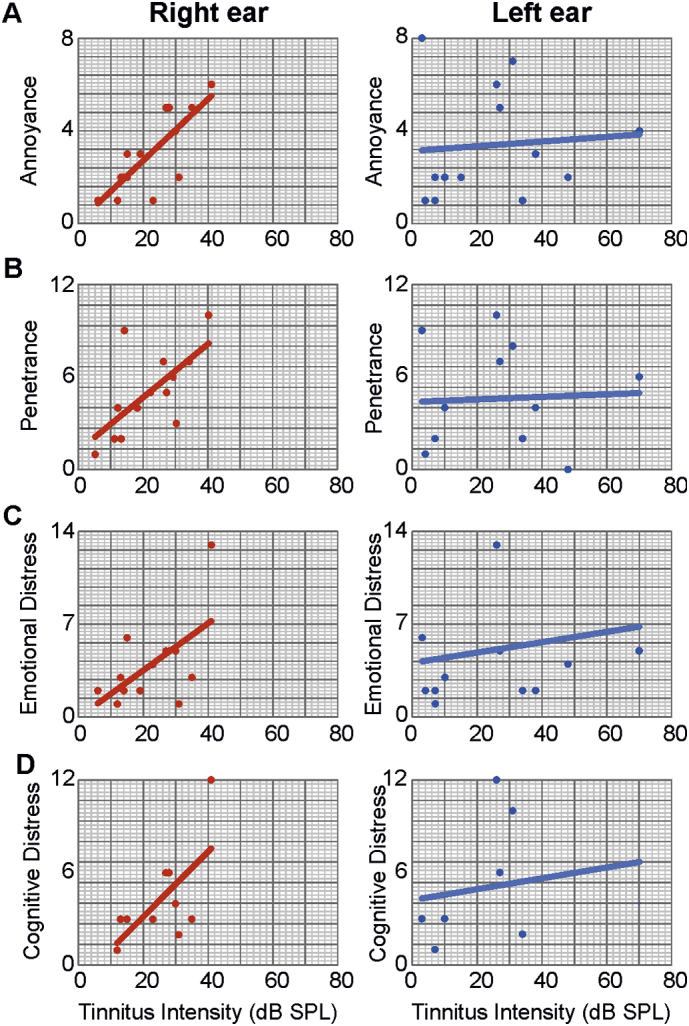
Tinnitus-Questionnaire sub-score correlation. (A) Correlation of tinnitus loudness (n depends on the perception of tinnitus) with the annoyance over the last days for right and left ears. n = 14 for the right ear, n=13 for the left ear. The tinnitus intensity is positively correlated with the annoyance over the last days score for the right ear (R = 0.58, *p* < 0.05) but not for the left ear (R = 0.18, *p* > 0.05). (B) Correlation of tinnitus loudness (n depends on the perception of tinnitus) with the tinnitus penetrance for right and left ears. n = 13 for the right ear, n = 11 for the left ear. The tinnitus intensity is positively correlated with the penetrance over the last days for the right ear (R = 0.77, *p* < 0.01) but not for the left ear (R = 0.28, *p* > 0.05). (C) Correlation of tinnitus loudness (n depends on the perception of tinnitus) with emotional distress for right and left ears. n = 13 for the right ear, n = 11 for the left ear. The tinnitus intensity is positively correlated with the emotional distress for the right ear (R = 0.84, *p* < 0.01) but not for the left ear (R = 0.22, *p* > 0.05) (D) Correlation of tinnitus loudness (n depends on the perception of tinnitus) with cognitive distress for right and left ears. n=10 for the right ear, n = 7 for the left ear. The tinnitus intensity is positively correlated with the cognitive distress for the right ear (R = 0.90, *p* < 0.01) but not for the left ear (R = 0.23, *p* > 0.05).

Remarkably, tinnitus loudness and the tinnitus score, reflected as annoyance, penetrance, emotional distress and cognitive distress (Supplementary Fig. S2A–E), correlated in the right (Fig. 2A, *R* *=* *0.68*, *p* *=* *0.004*) but not the left ear (Fig. 2A, *R* *=* *0.13*, *p* *=* *0.67*). Additionally, auditory perceptual difficulty was correlated with tinnitus loudness in the right ear (Fig. 2B, *R* *=* *0.79*, *p* *<* *0.05*) but not in the left ear (Fig. 2B, *R* *=* *0.25*, *p* *=* *0.58*), a surprising finding in light of the lack of an objective difference in the hearing thresholds. In general, we have to be careful with the results for the tinnitus questionnaire, depending on the small number; outliners can influence the results.

BERA was used to identify ABR wave I (generated by the distal peripheral portion of the auditory nerve) (Portmann et al., 1980), ABR wave III (generated by the spherical cells and globular cells of the cochlear nucleus projecting to the superior olivary complex (SOC)) (Melcher and Kiang, 1996), ABR wave V (generated by the medial superior olive and its projections to the nuclei in the lateral lemniscus and the inferior colliculus (IC)) and ABR wave VI (generated by IC output or the medial geniculate body (MGB)) (Moller et al., 1994) at stimulus level up to 75 dB SPL in the left and right ears.

The amplitude of ABR wave V was significantly reduced in the tinnitus group relative to that of the volunteer group at 75 dB SPL (Fig. 3A: three-way ANOVA with repetition: group *F* *=* *23.17*, *p* *<* *0.0001*; SPL *F* *=* *15.67*, *p* *<* *0.0001*; ear side *F* *=* *12.67*, *p* *=* *0.013*; group*SPL *F* *=* *4.32*, *p* *=* *0.035*; group*ear side *F* *=* *4.74*, *p* *=* *0.03*; ear side*SPL *F* *=* *7.58*, *p* *=* *0.021*; SPL*group*ear side *F* *=* *3.71*, *p* *=* *0.038*), while those of ABR waves I, III and VI did not differ significantly between the two groups. At the same stimulus intensity, the wave V latency exhibited a significant delay in the tinnitus group (Fig. 3B: three-way ANOVA with repetition: group *F* *=* *300.5*, *p* *<* *0.0001*; SPL *F* *=* *77.75*, *p* *<* *0.0001*; ear side *F* *=* *3.77*, *p* *=* *0.03*; group*SPL *F* *=* *6.22*, *p* *=* *0.025*; group*ear side *F* *=* *4.23*, *p* *=* *0.03*; ear side*SPL *F* *=* *5.84*, *p* *=* *0.029*; SPL*group*ear side *F* *=* *2.82*, *p* *=* *0.045*). While in general, women have higher ABR amplitudes than men (Hultcrantz et al., 2006), there was a reduction in the ABR amplitudes in tinnitus in both the male and female participants.

In summary, among the participants with and without tinnitus that did not differ in hearing thresholds, the tinnitus group showed a significantly reduced and delayed ABR wave V, a feature that would argue for reduced, rather than elevated, response gain in the ascending auditory pathway of the tinnitus group.

### 3.2 Reduced sound-evoked BOLD fMRI responses in auditory-specific regions in tinnitus

We aimed to test if reduced and delayed sound-induced brainstem responses are reflected in sound-evoked BOLD fMRI signals in anatomically predefined, auditory-specific ROIs (Table 1). BOLD fMRI signals were investigated in response to binaurally delivered (i) HF-chirp, (ii) LF-chirp, (iii) BB-chirp and (iv) rock music stimuli, using MRI-compatible headphones (Sensimetrics Model/S14). To correct for the variable TR, which could become obscured with cardiac gating during BOLD signal detection in the brainstem due to vascular pulsations (Guimaraes et al., 1998), we included a special TR-correction procedure (see Supplementary material). We identified significant activity levels using z-values from the statistical SPM analysis.

**Table 1:** Predefined ROIs for task evoked and resting-state fMRI.

A: Task evoked predefined ROIs				
Brain Region (Brodmann area)	MNI Coordinates			Radius
	X	Y	Z	
CN-R/CN-L	10/-10	-39	-45	3
SOC-R/SOC-L	13/-13	-35	-41	3
IC-R/IC-L	6/-6	-33	-11	3
MGB-R/MGB-L	18/-18	-24	1	3
BA41-R/BA41-L	46/-46	-25	7	3
BA42-R/BA42-L	64/-64	-22	9	3
BA22A-R/BA22A-L	54/-54	-6	-6	3
BA21A-R/BA21A-L	66/-66	-13	-5	3
BA22P-R/BA22P-L	67/-67	-27	3	3
BA21P-R/BA21P-L	66/-66	-22	-5	3
Hipp-R/Hipp-L	28/-28	-35	18	3
BA13P-R/BA13P-L	35/-35	-21	16	3
BA1-R/BA1-L	63/-63	-18	28	3
BA2-R/BA2-R	63/-63	-22	31	3
Mam.-Body-R/Mam.-Body-L	9/-9	-19	4	3


B: Resting-state predefined ROIs				
Brain Region (Brodmann area)	MNI Coordinates			Radius
	X	Y	Z	
CN-R/CN-L	10/-10	-39	-45	3
SOC-R/SOC-L	13/-13	-35	-41	3
IC-R/IC-L	6/-6	-33	-11	3
MGB-R/MGB-L	18/-18	-24	1	3
BA41-R/BA41-L	46/-46	-25	7	3
BA42-R/BA42-L	64/-64	-22	9	3
BA22A-R/BA22A-L	54/-54	-6	-6	3
BA21A-R/BA21A-L	66/-66	-13	-5	3
BA22P-R/BA22P-L	67/-67	-27	3	3
BA21P-R/BA21P-L	66/-66	-22	-5	3
Hipp-R/Hipp-L	28/-28	-35	18	3
BA13P-R/BA13P-L	35/-35	-21	16	3
BA13A-R/BA13A-L	36/-36	20	7	3
BA28-R/BA28-L	18/-18	-13	-22	3
Amyg-R/Amyg-L	22/-22	-5	-18	3
BA45-R/BA45-L	57/-57	26	14	3
BA46-R/BA46-L	46/-46	41	18	3
BA47-R/BA47-L	33/-33	32	-10	3
BA9M-R/BA9M-L	7/-7	50	30	3
BA9DL-R/BA9DL-L	50/-50	14	32	3

As one of the most striking observations, there was a significant reduction in evoked responses in the core Wernicke's reception/understanding system (Ardila et al., 2016), which includes the primary auditory cortex (AC) in Heschl's gyrus of the superior temporal gyrus Brodmann area 41 (BA41), the lateral superior temporal gyrus (BA42), the posterior superior temporal gyrus (BA22) and the multimodal region of BA21.

BOLD fMRI responses were reduced in the tinnitus group in BA41 and BA42 in response to BB stimuli (Table 2A, BA41 left (L)), HF (Table 2A, BA41 right (R) and BA42-R), LF (Table 2A, BA41-R) and rock music stimuli (Table 2A, BA41-L and BA42-R). In the tinnitus group, BA21 and BA22 also exhibited significantly reduced evoked responses to BB stimuli and to musical exposure compared to the volunteer group (Table 2A, BA21, BA22-L and -R). Neither of the Wernicke-related areas responded with elevated BOLD fMRI activity in the tinnitus group (Table 2B).

**Table 2:** Task evoked fMRI in tinnitus.

A: Reduced task evoked fMRI in defined ROIs		
Applied stimuli	Brain Region (Brodmann area)	Intensity (*Z*-score)
BB-Stimuli	BA41-L	-1.57
	BA21-L	-2.33
	BA22-L	-1.48
	BA21-R	-2.05
	BA22-R	-2.19
	MGB-L	-1.40
	Hippocampus-L	-1.51
	BA13P-L	-2.03
	Hippocampus-R	-1.80
HF-Stimuli	BA41-R	-1.76
	BA42-R	-2.33
	BA13P-R	-1.31
	Hippocampus-R	-1.78
	Hippocampus-L	-1.52
LF-Stimuli	BA41-R	-1.60
	BA13P-R	-1.42
Music-Stimuli	BA41-L	-1.40
	BA21-L	-2.37
	BA42-R	-1.99
	BA22-R	-2.24


B: Enhanced task evoked fMRI in defined ROIs		
Applied stimuli	Brain Region (Brodmann area)	Intensity (Z-score)
BB-Stimuli	BA13A-R	2.04
LF-Stimuli	BA1-L	1.74
	BA2-L	1.96
	BA13A-L	2.08
Music-Stimuli	BA13A-L	2.44
	Mam.-Body-R	2.20

The posterior insula (BA13P) is known to be responsible for sound detection (Sadaghiani et al., 2009), and the hippocampus is known to be responsible for shaping auditory skills (Kraus and White-Schwoch, 2015; Weinberger, 2015). Strikingly, both of these regions also reacted with reduced hemodynamic BOLD activity levels in response to the various sound stimuli, namely, BB (Table 2A, insula-L, BA13P-L, and hippocampus-L and -R), HF (Table 2A, insula-R and hippocampus-L, -R), and LF (Table 1A, insula-R). In addition, none of these regions responded with elevated BOLD activity (Table 2B).

In contrast, significantly elevated BOLD fMRI activity was found only in the anterior insula (BA13A) in response to BB (Table 2B, BA13A-R), LF (Table 2B, BA13A-L) and rock music (Table 2B, BA13A-R) stimuli and in the mammillary body in response to music stimuli (Table 2B, mammillary-body-R). The anterior insula, as a limbic-related cortex involved in distress (Wang et al., 2005), and the mammillary body, as part of the limbic system (Bubb et al., 2017), have both been described in previous studies to respond with elevated BOLD fMRI activity in tinnitus (Carpenter-Thompson et al., 2014; Vanneste et al., 2010). The primary somatosensory cortex was, interestingly, also among the few ROIs that responded with elevated BOLD fMRI activity in the tinnitus group (Table 2B, BA1-L, BA2-L), suggesting a loss of auditory-specific precision.

Following review of the potential correlations between the different observed events in the auditory brainstem in the tinnitus group, we noted that the reduced BOLD activity in the MGB region correlated significantly with the prolonged latency of ABR wave V, with higher significance on the right side than on the left side (BOLD activity MGB R with wave V latency right ear *p* *=* *0.0038, F* *=* *16.13*; BOLD activity MGB L with wave V latency in the left ear *p* *<* *0.05, F* *=* *4.1*, ANCOVA).

In conclusion: The tinnitus group showed significantly reduced sound-evoked BOLD activity in subcortical and cortical regions. This feature would argue for reduced, rather than elevated, response gain in the ascending auditory pathway in the tinnitus group.

### 3.3 Reduced positive resting-state connectivity in auditory-specific regions in tinnitus

Previous findings have suggested that task-evoked BOLD fMRI activity and behavioral performance are correlated with a higher level of synchronous positive fMRI activity at rest (Haag et al., 2015). To further specify the altered BOLD fMRI activity in the ascending auditory pathway in the tinnitus group, we specifically distinguished positive from negative r-fcMRI correlations. Positive r-fcMRI correlations are characterized by rather short time delays between signals, elevated synchronicity and balanced resting-state cerebral blood flow (rCBF) and resting-state cerebral blood volume (rCBV). Negative r-fcMRI correlations exhibit rather long time delays between signals with anti-correlated activity and occur possibly when rCBV is delayed, for example, compared to rCBF (Goelman et al., 2014; Lee et al., 2013).

In the first approach, whole-brain functional activities of predefined ROIs (Table 1) were compared between the participant group and the tinnitus group (Supplementary Table S3). Strikingly, significant reductions in the r-fcMRI responses of the right CN, bilateral SOC, bilateral IC, left MGB, and bilateral BA41/BA42 became evident in the tinnitus group in comparison to the volunteer group, while only a small region in the left BA42 exhibited enhanced r-fcMRI connectivity in the tinnitus group compared to the volunteer group (Supplementary Table S3).

We next specifically calculated the connectivities of defined ROIs (Table 1) within the auditory pathway of each volunteer and tinnitus group, namely, the CN, SOC, IC, MGB, BA41/42, and BA21/22, with those of regions related to (i) auditory processing and sound detection, such as BA13P and the hippocampus (Fig. 4); to (ii) the limbic and distress systems involved in fear and anxiety, particularly during tinnitus (Chen et al., 2017; Husain, 2016; Leaver et al., 2016a), including the amygdala, entorhinal cortex (BA28), and BA13A (Fig. 5); and to (iii) the attention/stress-regulating network, which, as part of the fronto-parietal or temporo-frontal network (Cieslik et al., 2015), comprises BA45, 46, and 47, dorsolateral BA9 (BA9DL) and medial BA9 (BA9M) (Fig. 6). It is important to note that the time course correlation analysis was considered significant only when the high Pearson correlation coefficient value and the *p*-value of the correlation was <0.05, with one out of three possibilities; (i) no correlation with (*p* *>* *0.05)*, (ii) positive connectivity (with a positive *R-*value and *p* *<* *0.05*), meaning that the two regions are functionally related, and (iii) negative connectivity (with a negative *R-*value and *p* *<* *0.05*), which is a significant but negative correlation (Goelman et al., 2014).

While positive connectivities between the homologous right and left hemisphere auditory brainstem, auditory midbrain and auditory cortex were observed in the volunteer group, the tinnitus group did not exhibit positive correlations between these regions (compare Figs. 4A and B, CN and SOC; IC, MGB, BA41, and BA42). At the same time, the significant positive connectivities seen in the volunteer group between the auditory brainstem nuclei, such as the CN, SOC and IC, and BA13P and the hippocampus were not detected in the tinnitus group (compare Figs. 4A and B, CN, SOC, and IC to BA13P-L and hippocampus-L) or were negatively correlated (Fig. 4B SOC and MGB to BA13P-R and hippocampus-R, -L).

We next observed that positive connectivities predominated between the primary and secondary AC regions and the emotional-distress regions, such as the amygdala, BA28 and BA13A but were not found to be positive in the tinnitus group (Figs. 5A, B, upper part, BA41, BA21/22 to amygdala, BA28 and BA13A). Instead, in the tinnitus group, significantly positive connectivities between these regions and lower auditory brainstem nuclei, such as the CN and the IC, were observed (Fig. 5B, upper part, IC, CN to amygdala, BA28 and BA13A).

The same was true for the positive connectivities seen between the primary and secondary AC regions and the temporofrontal network in the volunteer group. These positive connectivities were not observed in the tinnitus group (Fig. 6A, BA41/BA42 to BA45, BA47, and BA9), and instead, in this group, positive connectivities appeared between the temporofrontal network and lower auditory brainstem regions (Fig. 6B, IC, SOC, CN, MGB to BA45, BA47, and BA9). Notably, the pattern of positive connectivities between lower auditory brain regions and nodes of the emotional (Fig. 5B) or attention/stress-regulating (Fig. 6B) network in the tinnitus group mirrored, in some respects, those of the negative connectivities in the volunteer group (Fig. 6A, lower part, Fig. 6B, upper part, compare CN to BA9 or SOC to BA47, for example). We next specifically regarded connectivities in subregions of the prefrontal cortex as the medial prefrontal cortex (BA9M), which belongs to the default mode network and has been suggested to play a role in stress excitation (McKlveen et al., 2016; Utevsky et al., 2014). This region can be distinguished from the dorsolateral prefrontal cortex (BA9DL), which is essential for auditory-specific attentional tasks and is linked to stress inhibition (McKlveen et al., 2013; Rasgon et al., 2017). In the volunteer group, the primary and secondary AC regions were positively connected to BA9D-L, while the auditory nuclei, such as the CN, SOC, and MGB, were negatively connected to BA9M (Fig. 6A, compare the upper and lower panels). In the tinnitus group, in contrast, the CN and SOC were positively connected to BA9M, particularly on the right side (Supplementary Table S4), while the connectivity between the IC and BA9DL, for example, were instead negatively connected (Fig. 6B, compare the upper and lower panels). A trend (*p* *=* *0.08*) of a right-hemisphere dominance of the negative connectivity between lower auditory brainstem regions and temporofrontal attentional regions, such as BA45, BA47, and BA9M, in the volunteer group compared to the positive connectivity in the tinnitus group is shown in Supplementary Table S4.

Interestingly in this context, focusing on the changes in the auditory brainstem, we further observed that the reduced BB-chirp-induced BOLD fMRI response in the MGB in the tinnitus group (Table 2) was significantly correlated with more positive r-fcMRI connectivity of the MGB and BA9M, particularly in the right hemisphere (BOLD activity in the MGB-R correlated with that of the MGB-R/BA9M-R, *R* *=* *0.61, p* *<* *0.05, F* *=* *4*). Moreover the BB-chirp-induced BOLD activity in the right-hemisphere MGB (Table 2) correlated with that of the MGB-R/BA9M-L, *R* *=* *0.7*1, *p* *=* *0.002*, *F* *=* *11.5* or with the MGB-R/BA47-L, *R* *=* *0.68*, *p* *=* *0.04*, *F* *=* *4.68*, ANCOVA compared to the volunteer group.

Since the prefrontal cortex (PFC - BA9) has been found to control the HPA axis with right-hemisphere dominance (Sullivan and Gratton, 2002), we next compared cortisol levels at different times of the day in the volunteer and tinnitus group. We focused on salivary cortisol, as saliva is the most reliable body fluid for detecting HPA axis imbalances through cortisol levels (Elder et al., 2017; Verbeeten and Ahmet, 2017). The two-way ANOVA with repetition of the saliva samples did not show statistically significant differences between the volunteer and tinnitus groups in terms of salivary cortisol (Fig. 7; two-way ANOVA with repetition: time *F* *=* *118*, *p* *=* *0.0001*; group *F* *=* *3.7*, *p* *=* *0.062*; interaction *F* *=* *1.05*, *p* *=* *0.35*). Higher number of participants will be necessary to judge, if the tendency of increased salivary cortisol levels in the tinnitus group in comparison to the volunteer group (Fig. 7), may unravel an imbalance in the HPA axis.

## 4 Discussion

A reduced and delayed sound-induced auditory brainstem response that co-occurred with both reduced sound-evoked subcortical and cortical BOLD fMRI activity and with reduced interhemispheric r-fcMRI connectivities in auditory-specific regions was shown in this study in the tinnitus group. The findings are discussed in the context of a failure to increase central neural gain in tinnitus and a loss of context-specific recruitment of attentional cues due to an HPA axis imbalance.

### 4.1 Attenuation and delay of suprathreshold sound responsiveness in tinnitus

Although only participants with normal or mildly impaired hearing thresholds were included in the present study, we might expect differences in the hearing levels between 0 and 40 dB. In the present study, no significant differences in the hearing thresholds between participants with and without tinnitus were found. This finding argues for tinnitus being not primarily linked to hearing threshold deficits, as also shown in previous studies (Boyen et al., 2014; Geven et al., 2011; Gilles et al., 2016; Guest et al., 2017; Knipper et al., 2013). Independent if discordant damage of IHCs and OHCs (Jastreboff, 1990) or deafferentation causes the observed reduced and delayed ABR wave V in the tinnitus group (Fig. 2), elevated spontaneous activity levels in the lower brainstem regions may be the result as generally found in tinnitus of studies disregarding the origin of cochlear damage (Brozoski et al., 2002; Chen and Jastreboff, 1995; Kwon et al., 1999; Roberts et al., 2010). Previous models argued that the generation of the elevated spontaneous activity as explained in a computational model, for example, is the result of a preferential loss of low-SR, high-threshold fibers predicted to preferably correlate with a pathological increase in central gain and tinnitus (Schaette and McAlpine, 2011b), as otherwise the generation of a sufficient increase in the discharge rate to compensate for deprived auditory input may be hampered (Schaette and Kempter, 2009, Schaette and Kempter, 2012b). In contrast, we may argue that the reduced and delayed late ABR waves in the tinnitus group are possibly linked to deafferentation of high-SR AF, which exhibit low thresholds and at any given characteristic frequency exhibit the shortest latencies and define perceptual thresholds (Heil et al., 2008; Meddis, 2006). High-SR AF contribute to the threshold of the summed auditory nerve activity (Liberman and Kiang, 1978) and only when a critical degree is lost, would lower the threshold of the compound action potential threshold of the auditory fibers, reflected in ABR wave I (Bourien et al., 2014). Thereby, a differential degree of high-SR AF loss could explain the observation of reduced (Milloy et al., 2017) and unchanged (Paul et al., 2017, present findings) ABR wave I amplitude in tinnitus, as well as the differences in high-frequency hearing loss in tinnitus (Vielsmeier et al., 2015).

Elevated spontaneous activity can also be a response of imbalance or loss of dopaminergic efferent feedback to auditory fibers that when e.g. blocked reduce high-SR AF responses and elevate spontaneous firing rate (Ruel et al., 2001; Ruel et al., 2006). Thus, a discordant damage of inner and outer hair cell (feedback) responses (Baguley, 2002) may be also considered as an alternative source of reduced auditory-specific signaling, here observed in the tinnitus group.

While future studies should explicitly test this hypothesis, we conclude that the reduced and delayed ABR wave V in tinnitus described here can be explained by reduced discharge rates of auditory input, independent if caused through damage of IHCs synapses or discordant damage of IHCs and OHCs feedback responses.

### 4.2 Reduced sound-evoked BOLD fMRI responses in subcortical/cortical auditory nuclei in tinnitus

Strikingly, the present findings reported reduced sound-evoked BOLD fMRI activity in the AC (BA41, BA42, BA21, and BA22) and the MGB in participants with tinnitus, and the correlational analysis pointed to a relation between the latency of sound-induced late ABR wave V and sound-induced BOLD fMRI responses at the level of the MGB in the tinnitus group (Table 2). Reduced sound-evoked BOLD fMRI in the AC and MGB strongly supported the notion of reduced subcortical/cortical sound responsiveness in the tinnitus group compared to that in the volunteer group. This observation received indirect support through the reduced gray matter volumes observed in bilateral auditory cortical areas in the tinnitus group, as measured through voxel-based morphometry (VBM) (Schecklmann et al., 2013; Schneider et al., 2009). Any possible inconsistencies with previous studies may be explained by unmatched participants (Adjamian et al., 2009) or the co-occurrence with hyperacusis (Melcher et al., 2009). Other than in the AC, and in line with previous studies (Husain, 2016; Schmidt et al., 2017), reduced BOLD fMRI activity was also observed in the tinnitus group in regions involved in sound detection (Table 2), such as BA13P (said to improve sound detection thresholds through the direction of attentional resources toward audition (Sadaghiani et al., 2009)) and the hippocampus (important for accentuating behaviorally important sounds (Kilgard and Merzenich, 1998; Kraus and White-Schwoch, 2015; Weinberger, 2015). Lower and delayed auditory-specific flow may lead to reduced direction of attentional resources to less accentuated auditory stimuli, a hypothesis that would be strengthened by the elevated sound-induced BOLD fMRI activity in the somatosensory regions of BA1 and BA2 (Table 2) observed in the tinnitus group, which pointed to reduced auditory-specific processing. Future studies may challenge the hypothesis stating that a critical loss of driving force for the maintenance of auditory acuity can occur through the loss of high-SR AF. This would possibly reverse improved signal-to-noise ratios gained with the onset of hearing, as has been suggested in animal models (Chumak et al., 2016; Knipper et al., 2015; Schimmang et al., 2014). Loss of the signal-to-noise ratio would also explain predicted elevated noise levels in a tinnitus model (Zeng, 2013). Elevated noise levels are therefore here suggested as (i) the “pre‑tinnitus state” in the “Jastreboff Neurophysiological Model” (Jastreboff, 1999a; Jastreboff et al., 1996), (ii) the candidate correlates of the “tinnitus precursor” (Sedley et al., 2016) or (iii) the dysregulated resting state network between fronto-striatal and auditory-sensory regions in tinnitus (Leaver et al., 2016b). Within the tinnitus precursor model, perpetuation of tinnitus is predicted when focused attention increases the intensity of the tinnitus precursor that is normally ignored as imprecise evidence against the prevailing percept of “silence” (Sedley et al., 2016). In the “Jastreboff Neurophysiological Model” “negative emotional reinforcement”, described as fear, anxiety or tension, cause the limbic system to enhance and manifest pre-tinnitus activity that is typically ignored after habituation (Jastreboff, 1999a; Jastreboff et al., 1996). Hypothesizing, that the percept of silence is defined thresholds of high-SR auditory fibers (Heil et al., 2008; Meddis, 2006), a critical loss of high-SR AF as predicted here and in previous studies (Knipper et al., 2013; Rüttiger et al., 2013a; Singer et al., 2013a), might best explain the observed latency shifts and amplitude wave reductions in the tinnitus group. Important to note, that also discordant damage of inner and outer hair cells feedback responses (Baguley, 2002; Jastreboff, 1990) might cause discharge rate changes through e.g. imbalanced inhibition of auditory nerves and thus may underlie the observed reduced central sound responsiveness here observed in tinnitus patients.

The elevated somatosensory input in response to cochlear damage after hearing loss (Shore et al., 2008), the altered somatosensory-auditory integration in tinnitus (Crippa et al., 2010), as well as the elevated amplitude modulation thresholds in tinnitus patients (Paul et al., 2017) may be reconsidered in light of reduced auditory acuity in tinnitus.

### 4.3 Altered positive correlations in r-fcMRI between auditory-specific regions in tinnitus

While the BOLD signal provides information about neural activity on the basis of neurovascular coupling and increased local metabolism (Logothetis et al., 2001), a positively correlated, homogeneous hemodynamic BOLD signal during r-fcMRI reflects an accumulation of spontaneous, highly-synchronized, short time-lagged neural activations between two groups of neurons in two separate regions (Goelman et al., 2014). Negative connectivity is found in regions where a longer time lag between the time courses of the two correlated regions occurs. This delayed time course between two brain regions has been suggested to reflect a state of decreased synchrony or connectivity as a function of time (Goelman et al., 2014). Previously, more synchronous correlations at rest were linked to increased synchronicity of neural activity (evoked BOLD signals) and to better sensory performance (Haag et al., 2015). Regarding this aspect, the nearly absence of positive interhemispheric r-fcMRI connectivities between the auditory-specific nuclei, including the MGB and AC, of the tinnitus group (Fig. 5), the reduced fcMRI connectivities in auditory-specific ROIs of the tinnitus group detected at the whole-brain level in comparison to those of the volunteer group (Supplementary Table S3), and the reduced sound-evoked BOLD fMRI activity in the MGB and AC may be linked to and support that reduced sound-induced brainstem responses are associated with the tinnitus percept through reduced, rather than increased, central neural gain.

While previous studies described already reduced interhemispheric r-fcMRI connectivity among cortical regions in tinnitus patients (Kim et al., 2012; Lanting et al., 2014), the present study is the first to observe the decline in positive interhemispheric r-fcMRI connectivity, particularly in subcortical, lower-level auditory brainstem regions. Increased activity in higher order cortical areas and increased functional connectivity among them was previously observed to be linked to elevated speech comprehension in noise during childhood (Obleser et al., 2007), challenging tinnitus may be linked to partial reversal of this maturation process. Interestingly also, that reduced interhemispheric connectivity, mainly in the sensory system, was previously discussed as an internal convergence state that may precede depressive symptomatology (Ben-Shimol et al., 2015). In the present study, >90 % of the participants in the tinnitus group had scores that indicated low-grade tinnitus (Biesinger et al., 1998a; Goebel and Hiller, 1994; Zirke et al., 2010). These grades are typically recognized as not being yet linked to profound psychiatric comorbidities (Langguth et al., 2010). Thus, it will be interesting to investigate whether preceding depressive symptomatology can be detected through changes in interhemispheric connectivities already present in participants with low-grade tinnitus.

Moreover, in the tinnitus group, between the anterior insula (BA13A) and other regions involved in emotional distress (Fig. 5), as well between the temporofrontal attentional network (Fig. 6) hardly any positive connectivity to the AC were detected and instead these regions exhibited positive connectivity to the lower auditory brainstem regions.

Changes in the connectivities in attentional circuits (Husain, 2016; Schmidt et al., 2017), emotional-distress regions (Chen et al., 2017; Husain, 2016; Leaver et al., 2016b) or temporofrontal attentional networks (Leaver et al., 2016b; Rauschecker et al., 2015; Schlee et al., 2009; Schmidt et al., 2017; Vanneste and De Ridder, 2012) have been observed previously in patients with tinnitus; however, none of the previous studies examined lower auditory brainstem regions. Therefore, differences in the functional connectivities of the emotional-distress and temporofrontal attentional/stress-regulating regions to lower auditory brainstem nuclei, in tinnitus have escaped detection until now.

In this context, the altered connectivities to prefrontal cortex regions in tinnitus are remarkable. BA9 of the prefrontal cortex regulates the HPA axis stress response with a right-hemisphere dominance (Sullivan and Gratton, 2002). In the present study, the tinnitus group exhibited elevated positive connectivity of default mode network activity (BA9 M) (Fig. 6, Supplementary Table S4), a brain region said to be linked to stress excitation (McKlveen et al., 2016; McKlveen et al., 2013; Utevsky et al., 2014). This elevated r-fcMRI in BA9M correlated with the reduced sound-induced BOLD fMRI activity in the MGB. The reduced BOLD fMRI activity in the MGB, in turn, correlated with increased latencies of the ABR wave V responses. All of these events occurred with a trend of dominance in the right hemisphere, suggesting a possible causal relation between the observed right-hemisphere dominance of tinnitus loudness and the auditory percept in the group (Fig. 2, Supplementary Fig. S2). In this context, it is important to note that the auditory perceptual difficulty (defined through the Goebel-Hiller questionnaire score) is actually an estimate for central auditory processing and thus may be unrelated to hearing threshold.

It is interesting that greater negative connectivities of lower-level auditory brainstem regions to the BA9DL, a region suggested to inhibit stress (Shallice et al., 2008), were seen in tinnitus (Fig. 6, Supplementary Table S4). Elevated stress-enhancing BA9M connectivity and reduced stress-inhibiting BA9DL connectivity may thus be considered in future studies in the context of reported HPA axis imbalances observed in tinnitus (Hébert et al., 2012; Mazurek et al., 2012). Elevated stress-enhancing connectivity could also be the neural correlate of the previously predicted “negative emotional reinforcement” signal of the limbic system that according the “Jastreboff Neurophysiological Model” (Jastreboff, 1990) is essential to manifest the perception of pre-tinnitus activities.

HPA axis imbalances and glucocorticoid resistance lead to a loss of glucocorticoid-dependent metabolic support for behaviorally relevant perception when context-specific activity is reduced (de Kloet, 2014; Jeanneteau and Arango-Lievano, 2016). Previous predictions stating that the conscious perception of sound requires long-range connectivity between auditory and attention-related areas (de Lafuente and Romo, 2005) thus have to consider the risk of losing sound-specific perception and elevating phantom perception when energization involved in the recruitment of cortical regions that control attention under glucocorticoid influence are lost, and thereby noise is accentuated instead in the deprived circuit. These findings may provide an objective diagnostic advancement and may offer a new landmark for improving future clinical outcomes of intervention strategies for tinnitus. The observed reduced, rather than enhanced, auditory responsiveness here suggested as a neural correlate of tinnitus may need advanced sound stimulation as therapeutic intervention. In this context previous studies have shown that tinnitus therapeutic approaches, that aim to balance behavioral and emotional reactions to the tinnitus percept (Bauer et al., 2017; Jastreboff, 2015; Jastreboff et al., 1996; Seydel et al., 2015; Zenner et al., 2017) were equally effective upon usage of sound generators or open ear hearing aids. Both approaches provide identical, statistically indistinguishable results (Parazzini et al., 2011). This may motivate to validate the specificity and sensitivity of ABR waveform and fMRI resting state connectivity as objective markers in e.g. Tinnitus Retraining Therapy or in sound generator based therapies. In this context, future studies on participants with hyperacusis or other comorbidities are eligible.

The following are the supplementary data related to this article.

Supplementary Table S1 Study participants data. Supplementary Table S1

Supplementary Table S2 Study inclusion/exclusion requirements. Supplementary Table S2

Supplementary Table S3 Resting state fMRI. Supplementary Table S3

Supplementary Table S4 r-fcMRI - Auditory brainstem to temporofrontal attentional ROIs. Supplementary Table S4

Supplementary material Image 1

## Funding

This work was supported by the Deutsche Forschungsgemeinschaft DFG-Kni-316-4-1, SPP16-08 DFG and Hahn Stiftung (Index AG).

The ethics committee of Tübingen University (faculty of medicine) and University Hospital Tübingen (ethical approval-number 264-2016BO1) approved this study. Written informed consent was obtained from all participants at their first visit.

The authors declare no competing financial interests.

## References

[bb0005] Adjamian P., Sereda M., Hall D.A. (2009). The mechanisms of tinnitus: perspectives from human functional neuroimaging. Hear. Res..

[bb0010] Ardila A., Bernal B., Rosselli M. (2016). The language area of the brain: a functional reassessment. Rev. Neurol..

[bb0015] Baguley D.M. (2002). Mechanisms of tinnitus. Br. Med. Bull..

[bb0020] Bauer C.A., Berry J.L., Brozoski T.J. (2017). The effect of tinnitus retraining therapy on chronic tinnitus: a controlled trial. Laryngoscope Investig. Otolaryngol..

[bb0025] Ben-Shimol E., Gass N., Vollmayr B., Sartorius A., Goelman G. (2015). Reduced connectivity and inter-hemispheric symmetry of the sensory system in a rat model of vulnerability to developing depression. Neuroscience.

[bb0030] Biesinger E., Heiden C., Greimel V., Lendle T., Hoing R., Albegger K. (1998). HNO.

[bb0035] Biesinger E., Heiden C., Greimel V., Lendle T., Höing R., Albegger K. (1998). Strategien in Der Ambulaten Behandlung Des Tinnitus.

[bb0040] Bourien J., Tang Y., Batrel C., Huet A., Lenoir M., Ladrech S., Desmadryl G., Nouvian R., Puel J.L., Wang J. (2014). Contribution of auditory nerve fibers to compound action potential of the auditory nerve. J. Neurophysiol..

[bb0045] Boyen K., de Kleine E., van Dijk P., Langers D.R. (2014). Tinnitus-related dissociation between cortical and subcortical neural activity in humans with mild to moderate sensorineural hearing loss. Hear. Res..

[bb0050] Brozoski T.J., Bauer C.A., Caspary D.M. (2002). Elevated fusiform cell activity in the dorsal cochlear nucleus of chinchillas with psychophysical evidence of tinnitus. J. Neurosci..

[bb0055] Bubb E.J., Kinnavane L., Aggleton J.P. (2017). Hippocampal - diencephalic - cingulate networks for memory and emotion: an anatomical guide. Brain Neurosci. Adv..

[bb0060] Carpenter-Thompson J.R., Akrofi K., Schmidt S.A., Dolcos F., Husain F.T. (2014). Alterations of the emotional processing system may underlie preserved rapid reaction time in tinnitus. Brain Res..

[bb0065] Chen G.D., Jastreboff P.J. (1995). Salicylate-induced abnormal activity in the inferior colliculus of rats. Hear. Res..

[bb0070] Chen Y.C., Xia W., Chen H., Feng Y., Xu J.J., Gu J.P., Salvi R., Yin X. (2017). Tinnitus distress is linked to enhanced resting-state functional connectivity from the limbic system to the auditory cortex. Hum. Brain Mapp..

[bb0075] Chumak T., Rüttiger L., Lee S.C., Campanelli D., Zuccotti A., Singer W., Popelar J., Gutsche K., Geisler H.S., Schraven S.P., Jaumann M., Panford-Walsh R., Hu J., Schimmang T., Zimmermann U., Syka J., Knipper M. (2016). BDNF in lower brain parts modifies auditory fiber activity to gain fidelity but increases the risk for generation of central noise after injury. Mol. Neurobiol..

[bb0080] Cieslik E.C., Mueller V.I., Eickhoff C.R., Langner R., Eickhoff S.B. (2015). Three key regions for supervisory attentional control: evidence from neuroimaging meta-analyses. Neurosci. Biobehav. Rev..

[bb0085] Crippa A., Lanting C.P., van Dijk P., Roerdink J.B. (2010). A diffusion tensor imaging study on the auditory system and tinnitus. Open Neuroimaging J..

[bb0090] de Kloet E.R. (2014). From receptor balance to rational glucocorticoid therapy. Endocrinology.

[bb0095] de Lafuente V., Romo R. (2005). Neuronal correlates of subjective sensory experience. Nat. Neurosci..

[bb0100] Eggermont J.J. (2012). The Neuroscience of Tinnitus.

[bb0105] Eggermont J.J. (2015). Neural substrates of tinnitus in animal and human cortex: cortical correlates of tinnitus. HNO.

[bb0110] Eggermont J.J., Roberts L.E. (2012). The neuroscience of tinnitus: understanding abnormal and normal auditory perception. Front. Syst. Neurosci..

[bb0115] Eggermont J.J., Roberts L.E. (2015). Tinnitus: animal models and findings in humans. Cell Tissue Res..

[bb0120] Elder C.J., Harrison R.F., Cross A.S., Vilela R., Keevil B.G., Wright N.P., Ross R.J. (2017). Use of salivary cortisol and cortisone in the high- and low-dose synacthen test. Clin. Endocrinol..

[bb0125] Fischer A. (2013). Hyperakusis: Neues Screening-Instrument vorgestellt. HNO Nachrichten..

[bb0130] Geven L.I., de Kleine E., Free R.H., van Dijk P. (2011). Contralateral suppression of otoacoustic emissions in tinnitus patients. Otol. Neurotol..

[bb0135] Gilles A., Schlee W., Rabau S., Wouters K., Fransen E., Van de Heyning P. (2016). Decreased speech-in-noise understanding in young adults with tinnitus. Front. Neurosci..

[bb0140] Goebel G., Hiller W. (1994). The tinnitus questionnaire. A standard instrument for grading the degree of tinnitus. Results of a multicenter study with the tinnitus questionnaire. HNO.

[bb0145] Goelman G., Gordon N., Bonne O. (2014). Maximizing negative correlations in resting-state functional connectivity MRI by time-lag. PLoS One.

[bb0150] Gu J.W., Halpin C.F., Nam E.C., Levine R.A., Melcher J.R. (2010). Tinnitus, diminished sound-level tolerance, and elevated auditory activity in humans with clinically normal hearing sensitivity. J. Neurophysiol..

[bb0155] Guest H., Munro K.J., Prendergast G., Howe S., Plack C.J. (2017). Tinnitus with a normal audiogram: Relation to noise exposure but no evidence for cochlear synaptopathy. Hear. Res..

[bb0160] Guimaraes A.R., Melcher J.R., Talavage T.M., Baker J.R., Ledden P., Rosen B.R., Kiang N.Y., Fullerton B.C., Weisskoff R.M. (1998). Imaging subcortical auditory activity in humans. Hum. Brain Mapp..

[bb0165] Haag L.M., Heba S., Lenz M., Glaubitz B., Hoffken O., Kalisch T., Puts N.A., Edden R.A., Tegenthoff M., Dinse H., Schmidt-Wilcke T. (2015). Resting BOLD fluctuations in the primary somatosensory cortex correlate with tactile acuity. Cortex.

[bb0170] Hébert S., Canlon B., Hasson D. (2012). Emotional exhaustion as a predictor of tinnitus. Psychother. Psychosom..

[bb0175] Heil P., Neubauer H., Brown M., Irvine D.R. (2008). Towards a unifying basis of auditory thresholds: distributions of the first-spike latencies of auditory-nerve fibers. Hear. Res..

[bb0180] Hiller W., Goebel G., Rief W. (1994). Reliability of self-rated tinnitus distress and association with psychological symptom patterns. Br. J. Clin. Psychol..

[bb0185] Hultcrantz M., Simonoska R., Stenberg A.E. (2006). Estrogen and hearing: a summary of recent investigations. Acta Otolaryngol..

[bb0190] Husain F.T. (2016). Neural networks of tinnitus in humans: Elucidating severity and habituation. Hear. Res..

[bb0195] Jastreboff P.J. (1990). Phantom auditory perception (tinnitus): mechanisms of generation and perception. Neurosci. Res..

[bb0200] Jastreboff P.J., JWP H. (1999). The neurophysiological model of tinnitus and hyperacusis. Sixth International Tinnitus Seminar 1999, Cambridge.

[bb0205] Jastreboff P.J. (1999). Tinnitus retraining therapy. Br. J. Audiol..

[bb0210] Jastreboff P.J. (2015). 25 years of tinnitus retraining therapy. HNO.

[bb0215] Jastreboff P.J., Gray W.C., Gold S.L. (1996). Neurophysiological approach to tinnitus patients. Am. J. Otolaryngol..

[bb0220] Jeanneteau F., Arango-Lievano M. (2016). Linking mitochondria to synapses: new insights for stress-related neuropsychiatric disorders. Neural. Plast..

[bb0225] Johnson D.H., Kiang N.Y. (1976). Analysis of discharges recorded simultaneously from pairs of auditory nerve fibers. Biophys. J..

[bb0230] Kalappa B.I., Brozoski T.J., Turner J.G., Caspary D.M. (2014). Single unit hyperactivity and bursting in the auditory thalamus of awake rats directly correlates with behavioural evidence of tinnitus. J. Physiol..

[bb0235] Kaltenbach J.A., Zacharek M.A., Zhang J., Frederick S. (2004). Activity in the dorsal cochlear nucleus of hamsters previously tested for tinnitus following intense tone exposure. Neurosci. Lett..

[bb0240] Kilgard M.P., Merzenich M.M. (1998). Cortical map reorganization enabled by nucleus basalis activity. Science.

[bb0245] Kim J.Y., Kim Y.H., Lee S., Seo J.H., Song H.J., Cho J.H., Chang Y. (2012). Alteration of functional connectivity in tinnitus brain revealed by resting-state fMRI? A pilot study. Int. J. Audiol..

[bb0250] Knipper M., Van Dijk P., Nunes I., Rüttiger L., Zimmermann U. (2013). Advances in the neurobiology of hearing disorders: recent developments regarding the basis of tinnitus and hyperacusis. Prog. Neurobiol..

[bb0255] Knipper M., Panford-Walsh R., Singer W., Rüttiger L., Zimmermann U. (2015). Specific synaptopathies diversify brain responses and hearing disorders: you lose the gain from early life. Cell Tissue Res..

[bb0260] Kraus N., White-Schwoch T. (2015). Unraveling the Biology of Auditory Learning: a Cognitive-Sensorimotor-reward Framework. Trends Cogn. Sci..

[bb0265] Kwon O., Jastreboff M.M., Hu S., Shi J., Pj J. (1999). Modification of single-unit activity related to noise-induced tinnitus in rats. Proceedings of the Sixth International Tinnitus Seminar.

[bb0270] Langguth B., Landgrebe M., Kleinjung T., Strutz J., Hajak G. (2010). Tinnitus and psychiatric comorbidities. HNO.

[bb0275] Lanting C.P., de Kleine E., Langers D.R., van Dijk P. (2014). Unilateral tinnitus: changes in connectivity and response lateralization measured with FMRI. PLoS One.

[bb0280] Leaver A.M., Seydell-Greenwald A., Rauschecker J.P. (2016). Auditory-limbic interactions in chronic tinnitus: challenges for neuroimaging research. Hear. Res..

[bb0285] Leaver A.M., Turesky T.K., Seydell-Greenwald A., Morgan S., Kim H.J., Rauschecker J.P. (2016). Intrinsic network activity in tinnitus investigated using functional MRI. Hum. Brain Mapp..

[bb0290] Lee M.H., Smyser C.D., Shimony J.S. (2013). Resting-state fMRI: a review of methods and clinical applications. AJNR Am. J. Neuroradiol..

[bb0295] Liberman M.C., Kiang N.Y. (1978). Acoustic trauma in cats. Cochlear pathology and auditory-nerve activity. Acta Otolaryngol. Suppl..

[bb0300] Logothetis N.K., Pauls J., Augath M., Trinath T., Oeltermann A. (2001). Neurophysiological investigation of the basis of the fMRI signal. Nature.

[bb0305] Marks K.L., Martel D.T., Wu C., Basura G.J., Roberts L.E., Schvartz-Leyzac K.C., Shore S.E. (2018). Auditory-somatosensory bimodal stimulation desynchronizes brain circuitry to reduce tinnitus in Guinea pigs and humans. Sci. Transl. Med..

[bb0310] Mazurek B., Haupt H., Olze H., Szczepek A.J. (2012). Stress and tinnitus-from bedside to bench and back. Front. Syst. Neurosci..

[bb0315] McKlveen J.M., Myers B., Flak J.N., Bundzikova J., Solomon M.B., Seroogy K.B., Herman J.P. (2013). Role of prefrontal cortex glucocorticoid receptors in stress and emotion. Biol. Psychiatry.

[bb0320] McKlveen J.M., Morano R.L., Fitzgerald M., Zoubovsky S., Cassella S.N., Scheimann J.R., Ghosal S., Mahbod P., Packard B.A., Myers B., Baccei M.L., Herman J.P. (2016). Chronic stress increases prefrontal inhibition: a mechanism for stress-induced prefrontal dysfunction. Biol. Psychiatry.

[bb0325] Meddis R. (2006). Auditory-nerve first-spike latency and auditory absolute threshold: a computer model. J. Acoust. Soc. Am..

[bb0330] Melcher J.R., Kiang N.Y. (1996). Generators of the brainstem auditory evoked potential in cat. III: Identified cell populations. Hear. Res..

[bb0335] Melcher J.R., Levine R.A., Bergevin C., Norris B. (2009). The auditory midbrain of people with tinnitus: abnormal sound-evoked activity revisited. Hear. Res..

[bb0340] Milloy V., Fournier P., Benoit D., Norena A., Koravand A. (2017). Auditory brainstem responses in tinnitus: a review of who, how, and what?. Front. Aging Neurosci..

[bb0345] Moller A.R., Jannetta P.J., Jho H.D. (1994). Click-evoked responses from the cochlear nucleus: a study in human. Electroencephalogr. Clin. Neurophysiol..

[bb0350] Mumford J.A. (2012). A power calculation guide for fMRI studies. Soc. Cogn. Affect. Neurosci..

[bb0355] Noreña A.J. (2015). Revisiting the cochlear and central mechanisms of tinnitus and therapeutic approaches. Audiol. Neurootol..

[bb0360] Norena A.J., Eggermont J.J. (2003). Changes in spontaneous neural activity immediately after an acoustic trauma: implications for neural correlates of tinnitus. Hear. Res..

[bb0365] Obleser J., Wise R.J., Dresner M.A., Scott S.K. (2007). Functional integration across brain regions improves speech perception under adverse listening conditions. J. Neurosci..

[bb0370] Parazzini M., Del Bo L., Jastreboff M., Tognola G., Ravazzani P. (2011). Open ear hearing aids in tinnitus therapy: an efficacy comparison with sound generators. Int. J. Audiol..

[bb0375] Paul B.T., Bruce I.C., Roberts L.E. (2017). Evidence that hidden hearing loss underlies amplitude modulation encoding deficits in individuals with and without tinnitus. Hear. Res..

[bb0380] Portmann M., Cazals Y., Negrevergne M., Aran J.M. (1980). Transtympanic and surface recordings in the diagnosis of retrocochlear disorders. Acta Otolaryngol..

[bb0385] Rasgon A., Lee W.H., Leibu E., Laird A., Glahn D., Goodman W., Frangou S. (2017). Neural correlates of affective and non-affective cognition in obsessive compulsive disorder: a meta-analysis of functional imaging studies. Eur. Psychiatry.

[bb0390] Rauschecker J.P., May E.S., Maudoux A., Ploner M. (2015). Frontostriatal Gating of Tinnitus and Chronic Pain. Trends Cogn. Sci..

[bb0395] Roberts L.E., Eggermont J.J., Caspary D.M., Shore S.E., Melcher J.R., Kaltenbach J.A. (2010). Ringing ears: the neuroscience of tinnitus. J. Neurosci..

[bb0400] Ruel J., Nouvian R., Gervais d'Aldin C., Pujol R., Eybalin M., Puel J.L. (2001). Dopamine inhibition of auditory nerve activity in the adult mammalian cochlea. Eur. J. Neurosci..

[bb0405] Ruel J., Wang J., Dememes D., Gobaille S., Puel J.L., Rebillard G. (2006). Dopamine transporter is essential for the maintenance of spontaneous activity of auditory nerve neurones and their responsiveness to sound stimulation. J. Neurochem..

[bb0410] Rüttiger L., Singer W., Panford-Walsh R., Matsumoto M., Lee S.C., Zuccotti A., Zimmermann U., Jaumann M., Rohbock K., Xiong H., Knipper M. (2013). The reduced cochlear output and the failure to adapt the central auditory response causes tinnitus in noise exposed rats. PLoS One.

[bb0415] Rüttiger L., Singer W., Panford-Walsh R., Matsumoto M., Lee S.C., Zuccotti A., Zimmermann U., Jaumann M., Rohbock K., Xiong H., Knipper M. (2013). The reduced cochlear output and the failure to adapt the central auditory response causes tinnitus in noise exposed rats. PLoS One.

[bb0420] Ruttiger L., Zimmermann U., Knipper M. (2017). Biomarkers for hearing dysfunction: facts and outlook. ORL J. Otorhinolaryngol. Relat. Spec..

[bb0425] Sadaghiani S., Hesselmann G., Kleinschmidt A. (2009). Distributed and antagonistic contributions of ongoing activity fluctuations to auditory stimulus detection. J. Neurosci..

[bb0430] Schaette R., Kempter R. (2009). Predicting tinnitus pitch from patients' audiograms with a computational model for the development of neuronal hyperactivity. J. Neurophysiol..

[bb0435] Schaette R., Kempter R. (2012). Computational models of neurophysiological correlates of tinnitus. Front. Syst. Neurosci..

[bb0440] Schaette R., Kempter R. (2012). Computational models of neurophysiological correlates of tinnitus. Front. Syst. Neurosci..

[bb0445] Schaette R., McAlpine D. (2011). Tinnitus with a normal audiogram: physiological evidence for hidden hearing loss and computational model. J. Neurosci..

[bb0450] Schaette R., McAlpine D. (2011). Tinnitus with a normal audiogram: physiological evidence for hidden hearing loss and computational model. J. Neurosci..

[bb0455] Schecklmann M., Lehner A., Poeppl T.B., Kreuzer P.M., Rupprecht R., Rackl J., Burger J., Frank E., Hajak G., Langguth B., Landgrebe M. (2013). Auditory cortex is implicated in tinnitus distress: a voxel-based morphometry study. Brain Struct. Funct..

[bb0460] Schimmang T., Duran Alonso B., Zimmermann U., Knipper M. (2014). Is there a relationship between brain-derived neurotrophic factor for driving neuronal auditory circuits with onset of auditory function and the changes following cochlear injury or during aging?. Neuroscience.

[bb0465] Schlee W., Mueller N., Hartmann T., Keil J., Lorenz I., Weisz N. (2009). Mapping cortical hubs in tinnitus. BMC Biol..

[bb0470] Schmidt S.A., Carpenter-Thompson J., Husain F.T. (2017). Connectivity of precuneus to the default mode and dorsal attention networks: a possible invariant marker of long-term tinnitus. Neuroimage Clin..

[bb0475] Schneider P., Andermann M., Wengenroth M., Goebel R., Flor H., Rupp A., Diesch E. (2009). Reduced volume of Heschl's gyrus in tinnitus. NeuroImage.

[bb0480] Sedley W., Friston K.J., Gander P.E., Kumar S., Griffiths T.D. (2016). An integrative tinnitus model based on sensory precision. Trends Neurosci..

[bb0485] Seydel C., Haupt H., Szczepek A.J., Hartmann A., Rose M., Mazurek B. (2015). Three years later: report on the state of well-being of patients with chronic tinnitus who underwent modified tinnitus retraining therapy. Audiol. Neurootol..

[bb0490] Shallice T., Stuss D.T., Picton T.W., Alexander M.P., Gillingham S. (2008). Mapping task switching in frontal cortex through neuropsychological group studies. Front. Neurosci..

[bb0495] Shore S.E., Koehler S., Oldakowski M., Hughes L.F., Syed S. (2008). Dorsal cochlear nucleus responses to somatosensory stimulation are enhanced after noise-induced hearing loss. Eur. J. Neurosci..

[bb0500] Shore S.E., Roberts L.E., Langguth B. (2016). Maladaptive plasticity in tinnitus—triggers, mechanisms and treatment. Nat. Rev. Neurol..

[bb0505] Singer W., Zuccotti A., Jaumann M., Lee S.C., Panford-Walsh R., Xiong H., Zimmermann U., Franz C., Geisler H.S., Köpschall I., Rohbock K., Varakina K., Verpoorten S., Reinbothe T., Schimmang T., Rüttiger L., Knipper M. (2013). Noise-induced inner hair cell ribbon loss disturbs central arc mobilization: a novel molecular paradigm for understanding tinnitus. Mol. Neurobiol..

[bb0510] Singer W., Zuccotti A., Jaumann M., Lee S.C., Panford-Walsh R., Xiong H., Zimmermann U., Franz C., Geisler H.S., Köpschall I., Rohbock K., Varakina K., Verpoorten S., Reinbothe T., Schimmang T., Rüttiger L., Knipper M. (2013). Noise-induced inner hair cell ribbon loss disturbs central arc mobilization: a novel molecular paradigm for understanding tinnitus. Mol. Neurobiol..

[bb0515] Singer W., Kasini K., Manthey M., Eckert P., Armbruster P., Vogt M., Jaumann M., Dotta M., Yamahara K., Harasztosi C., Zimmermann U., Knipper M., Rüttiger L. (2018). The glucocorticoid antagonist mifepristone attenuates sound-induced long-term deficits in auditory nerve response and central auditory processing in female rats. FASEB J..

[bb0520] Song J.J., De Ridder D., Weisz N., Schlee W., Van de Heyning P., Vanneste S. (2014). Hyperacusis-associated pathological resting-state brain oscillations in the tinnitus brain: a hyperresponsiveness network with paradoxically inactive auditory cortex. Brain Struct. Funct..

[bb0525] Sullivan R.M., Gratton A. (2002). Prefrontal cortical regulation of hypothalamic-pituitary-adrenal function in the rat and implications for psychopathology: side matters. Psychoneuroendocrinology.

[bb0530] Utevsky A.V., Smith D.V., Huettel S.A. (2014). Precuneus is a functional core of the default-mode network. J. Neurosci..

[bb0535] Vanneste S., De Ridder D. (2012). The involvement of the left ventrolateral prefrontal cortex in tinnitus: a TMS study. Exp. Brain Res..

[bb0540] Vanneste S., Plazier M., der Loo E., de Heyning P.V., Congedo M., De Ridder D. (2010). The neural correlates of tinnitus-related distress. NeuroImage.

[bb0545] Verbeeten K.C., Ahmet A.H. (2017). The role of corticosteroid-binding globulin in the evaluation of adrenal insufficiency. J. Pediatr. Endocrinol. Metab..

[bb0550] Vielsmeier V., Lehner A., Strutz J., Steffens T., Kreuzer P.M., Schecklmann M., Landgrebe M., Langguth B., Kleinjung T. (2015). The relevance of the high frequency audiometry in tinnitus patients with normal hearing in conventional pure-tone audiometry. Biomed. Res. Int..

[bb0555] Wang J., Rao H., Wetmore G.S., Furlan P.M., Korczykowski M., Dinges D.F., Detre J.A. (2005). Perfusion functional MRI reveals cerebral blood flow pattern under psychological stress. Proc. Natl. Acad. Sci. U. S. A..

[bb0560] Weinberger N.M. (2015). New perspectives on the auditory cortex: learning and memory. Handb. Clin. Neurol..

[bb0565] Weisz N., Moratti S., Meinzer M., Dohrmann K., Elbert T. (2005). Tinnitus perception and distress is related to abnormal spontaneous brain activity as measured by magnetoencephalography. PLoS Med..

[bb0570] Weisz N., Muller S., Schlee W., Dohrmann K., Hartmann T., Elbert T. (2007). The neural code of auditory phantom perception. J. Neurosci..

[bb0575] Yang S., Bao S. (2013). Homeostatic mechanisms and treatment of tinnitus. Restor. Neurol. Neurosci..

[bb0580] Yang S., Weiner B.D., Zhang L.S., Cho S.J., Bao S. (2011). Homeostatic plasticity drives tinnitus perception in an animal model. Proc. Natl. Acad. Sci. U. S. A..

[bb0585] Zeng F.G. (2013). An active loudness model suggesting tinnitus as increased central noise and hyperacusis as increased nonlinear gain. Hear. Res..

[bb0590] Zenner H.P., Delb W., Kroner-Herwig B., Jager B., Peroz I., Hesse G., Mazurek B., Goebel G., Gerloff C., Trollmann R., Biesinger E., Seidler H., Langguth B. (2017). A multidisciplinary systematic review of the treatment for chronic idiopathic tinnitus. Eur. Arch. Otorhinolaryngol..

[bb0595] Zirke N., Goebel G., Mazurek B. (2010). HNO.

