# Supplementary material for: Reduced sound-evoked and resting-state BOLD fMRI connectivity in tinnitus
Source: Neuroimage Clin. 2018 Aug 31;20:637–49. doi: 10.1016/j.nicl.2018.08.029 (PMC6128096; doi:10.1016/j.nicl.2018.08.029)
Supplement: Supplementary Table S1 — Study participants data. [file mmc1.docx]

| **Supplementary Table 1. Study participants data** | | | | | | | | | | | | | | | | | | |  |  |
| --- | --- | --- | --- | --- | --- | --- | --- | --- | --- | --- | --- | --- | --- | --- | --- | --- | --- | --- | --- | --- |
| **A:** Study participants | | | | | | | | | | | | | | | | | | |  |  |
| Volunteer | | | | | | | | | | | Tinnitus | | | | | | | |  |  |
| Volunteer | | | | Age | | | Sex | | Handedness | | Tinnitus | Age | | Sex | | | Handedness | | | |
| KN20 | | | | 27 | | | male | | right | | T001 | 36 | | male | | | left | | | |
| KN24 | | | | 28 | | | male | | right | | T002 | 21 | | male | | | right | | | |
| KN15 | | | | 27 | | | male | | right | | T003 | 31 | | male | | | right | | | |
| KN05 | | | | 41 | | | female | | left | | T006 | 45 | | female | | | right | | | |
| KN23 | | | | 22 | | | male | | right | | T007 | 61 | | male | | | right | | | |
| KN16 | | | | 27 | | | male | | left | | T009 | 34 | | male | | | right | | | |
| KN09 | | | | 19 | | | male | | right | | TN01 | 26 | | male | | | right | | | |
| K003 | | | | 53 | | | female | | right | | TN02 | 31 | | female | | | left | | | |
| KN03 | | | | 18 | | | male | | right | | TN03 | 34 | | male | | | left | | | |
| K006 | | | | 39 | | | male | | right | | TN05 | 33 | | male | | | right | | | |
| KN19 | | | | 53 | | | male | | right | | TN07 | 49 | | male | | | right | | | |
| KN22 | | | | 26 | | | male | | right | | TN08 | 27 | | male | | | right | | | |
| KN11 | | | | 24 | | | female | | right | | TN10 | 25 | | female | | | right | | | |
| KN01 | | | | 21 | | | female | | right | | TN11 | 25 | | female | | | right | | | |
| KN21 | | | | 28 | | | male | | right | | TN16 | 26 | | male | | | left | | | |
| K004 | | | | 59 | | | female | | right | | TN18 | 61 | | female | | | right | | | |
| K001 | | | | 52 | | | female | | right | | TN26 | 56 | | female | | | right | | | |
| **B:** Study participants (tinnitus) | | | | | | | | | | | | | | | | | | | | |
|  |  | |  | | Tinnitus laterality | | | | | | | | Tinnitus frequency & Intensity | | | | | | |  |
| Tinnitus | | Tinnitus Score | | | | Right | | Left | | Both Sides Inner Head | | | Right | | | Left | | | |  |
|  |  |  |  |  |  |  |  |  |  |  |  |  | Hz | | dB | Hz | | dB | |  |
| T001 | | 0 | | | | low | | moderate | | moderate | | | 8000 | | 15 | 8000 | | 15 | |  |
| T002 | | 3 | | | | low | | low | | ‐‐‐ | | | 10.000 | | 6 | 10.000 | | 4 | |  |
| T003 | | 17 | | | | low | | low | | ‐‐‐ | | | 6000 | | 30 | 6000 | | 70 | |  |
| T006 | | 21 | | | | moderate | | moderate | | ‐‐‐ | | | 6000 | | 27 | 6000 | | 27 | |  |
| T007 | | 10 | | | | low | | ‐‐‐ | | ‐‐‐ | | | 4000 | | 23 | ‐‐‐ | | ‐‐‐ | |  |
| T009 | | 7 | | | | ‐‐‐ | | moderate | | ‐‐‐ | | | ‐‐‐ | | ‐‐‐ | 8000 | | 34 | |  |
| TN01 | | 24 | | | | moderate | | ‐‐‐ | | ‐‐‐ | | | 10.000 | | 35 | ‐‐‐ | | ‐‐‐ | |  |
| TN02 | | 7 | | | | low | | ‐‐‐ | | ‐‐‐ | | | 8000 | | 31 | ‐‐‐ | | ‐‐‐ | |  |
| TN03 | | 48 | | | | ‐‐‐ | | low | | ‐‐‐ | | | ‐‐‐ | | ‐‐‐ | 10.000 | | 31 | |  |
| TN05 | | 12 | | | | very low | | inaudible | | ‐‐‐ | | | 10.000 | | 28 | ‐‐‐ | | ‐‐‐ | |  |
| TN07 | | 4 | | | | ‐‐‐ | | ‐‐‐ | | mild | | | 10.000 | | 47 | 10.000 | | 48 | |  |
| TN08 | | 13 | | | | inaudible | | inaudible | | very low | | | 10.000 | | 13 | 10.000 | | 10 | |  |
| TN10 | | 7 | | | | inaudible | | inaudible | | inaudible | | | 4000 | | 12 | 8000 | | 7 | |  |
| TN11 | | 10 | | | | very low | | very low | | very low | | | 6000 | | 14 | 8000 | | 7 | |  |
| TN16 | | 19 | | | | high | | low | | moderate | | | 8000 | | 15 | 6000 | | 3 | |  |
| TN18 | | 44 | | | | moderate | | high | | moderate | | | 4000 | | 41 | 3000 | | 26 | |  |
| TN26 | | 6 | | | | low | | moderate | | ‐‐‐ | | | 6000 | | 19 | 6000 | | 38 | |  |
