# Supplementary material for: Reduced sound-evoked and resting-state BOLD fMRI connectivity in tinnitus
Source: Neuroimage Clin. 2018 Aug 31;20:637–49. doi: 10.1016/j.nicl.2018.08.029 (PMC6128096; doi:10.1016/j.nicl.2018.08.029)
Supplement: Supplementary Table S2 — Study inclusion/exclusion requirements. [file mmc2.docx]

| **Supplementary Table 2. Study inclusion/exclusion requirements** | | | |
| --- | --- | --- | --- |
| Study inclusion criteria | | | |
| Volunteer: | | | Tinnitus: |
| - No tinnitus suffering at the time of examination or as a medical history | | | - History of continuous tinnitus of more than 4 weeks |
|  | | | - Tinnitus in one or both sides |
|  | - PTA with low-grade hearing loss at a hearing threshold up to 40 dB - Clinically free microscopic ear examination and normal eardrum mobility - Age >18 to <70 years - Signed consent form. Written and oral information, which takes place  on request in the native language of the subjects | | |
| Study exclusion criteria for tinnitus and non-tinnitus subjects | | | |
| 1. Immune-suppressive drugs (*e.g.* daily cortisone) 2. Pulsatile tinnitus 3. Intermittent non-persistent tinnitus 4. Retro-cochlear hearing loss (as detected by ABR) 5. Hearing aid 6. Vertigo 7. Acoustic trauma 8. Diabetes mellitus type I and type II 9. Pregnancy 10. Drug therapy for tinnitus in the last 4 weeks 11. Ear surgery (*e.g.* tympanoplasty, middle-ear implants) 12. Tinnitus as a secondary symptom underlying another disease (*e.g.* acoustic neuroma or drug tinnitus) 13. Menière disease, endolymphatic hydrops 14. Unilateral or bilateral deafness 15. History of years of noise exposure 16. History of craniocerebral trauma (grade II/III) 17. Cervicogenic or jaw-related tinnitus 18. Conductive hearing with a hearing loss of more than 10 dB at more than 2 frequencies 19. Chronic ear canal or middle-ear infections 20. History of epilepsy suffering, Parkinson's disease and/or dementing illness 21. History of concomitant treatment for neurological and psychiatric disorders (*e.g.* Schizophrenia, depression) 22. Drug or alcohol dependency 23. Renal impairment with an increased creatinine (>160 mol/l = 1.8 mg/dl) 24. Currently in treatment due to a cancerous condition (*e.g.* leukemia) 25. Fear of closed spaces (claustrophobia) 26. Clinical history of cardiovascular disease (severe coronary heart disease) 27. Clinical history restricted temperature sensation and/or increased sensitivity to heating of the body 28. Incapacitated subjects 29. Subjects whom their German is not sufficiently powerful to the instructions to be understood in the context of the study and the questions of the Tinnitus Questionnaire 30. Permanent metal parts in or on the body (pacemakers, artificial heart valves), metal prostheses implanted, magnetic metal parts (screws, plates of operations), spiral, metal fragments/shrapnel, fixed braces, acupuncture needle, insulin pump, intraport, large tattoos) 31. Contrast agent allergy or hypersensitivity to contrast agents 32. Treatments of tinnitus maskers, noiser, hyperbaric oxygen or acupuncture <4 weeks | | | |
| Study medications | | | |
| Investigational Medications: | | In this case-control study, no drug candidate will be tested | |
| Non-Investigational Medications: | | | |
| - Permitted concomitant medications | | - Each existing therapy since 4 weeks basic independent of a tinnitus disease to which the patient is already set and not an exclusion criterion must be maintained, for example, β-blockers in hypertension | |
|  | | - Some painkillers (paracetamol, ibuprofen) as needed | |
| - Disallowed concomitant medications | | - Central-acting drugs (neuroleptics, haloperidol, L-dopa) | |
|  |  | - Drugs used to improve the circulation (pentoxyfilline) | |
|  |  | - Masker, noiser | |
|  |  | - Hyperbaric oxygen therapy (HBO) | |
|  |  | - Acupuncture | |
|  |  | - Autogenous training and psychotherapy | |
|  |  | - N.B. Treatments of tinnitus-like maskers, noiser, hyperbaric oxygen, or acupunc­ture must have been completed prior to the study at least 4 weeks | |
