# Supplementary material for: Reduced sound-evoked and resting-state BOLD fMRI connectivity in tinnitus
Source: Neuroimage Clin. 2018 Aug 31;20:637–49. doi: 10.1016/j.nicl.2018.08.029 (PMC6128096; doi:10.1016/j.nicl.2018.08.029)
Supplement: Supplementary Table S3 — Resting state fMRI. [file mmc3.docx]

| **Supplementary Table 3. Resting state fMRI** | |
| --- | --- |
| **A:** Reduced r-fMRI in the tinnitus group | |
| Brain Region  (Brodmann Area) | Intensity (Z-score) |
| CN R | -3.72 |
| SOC R | -8.42 |
| SOC L | -7.99 |
| IC R | -6.53 |
| IC L | -7.18 |
| MGB L | -1.92 |
| BA41 R | -4.11 |
| BA42 R | -5.04 |
| BA41 L | -6.62 |
| BA42 L | -4.59 |
| **B:** Enhanced r-fMRI in the tinnitus group | |
| Brain Region  (Brodmann Area) | Intensity (Z-score) |
| BA42 L | 3.23 |
