# Supplementary material for: Reduced sound-evoked and resting-state BOLD fMRI connectivity in tinnitus
Source: Neuroimage Clin. 2018 Aug 31;20:637–49. doi: 10.1016/j.nicl.2018.08.029 (PMC6128096; doi:10.1016/j.nicl.2018.08.029)
Supplement: Supplementary Table S4 — r-fcMRI - Auditory brainstem to temporofrontal attentional ROIs. [file mmc4.docx]

| **SupplementaryTable 4. r-fcMRI – Auditory brainstem to temporofrontal attentional ROIs** | | | | | | | | | | | | | | | | | | |
| --- | --- | --- | --- | --- | --- | --- | --- | --- | --- | --- | --- | --- | --- | --- | --- | --- | --- | --- |
| Volunteer | | | | | | | | | | | | | | | | | | |
| Right | | | | | | | | Left | | | | | | | | | | |
| RR | | | | RL | | | | | LR | | | | | LL | | | | |
|  |  |  |  |  |  |  |  | | |  |  |  |  | |  |  |  |  |
| ROI |  | ROI | r | ROI |  | ROI | r | | | ROI |  | ROI | r | | ROI |  | ROI | r |
| SOC-R | | BA45-R | -0,5078 | CN-R | | BA9M-L | -0,5245 | | | SOC-L | | BA45-R | -0,5078 | | MGB-L | | BA9M-L | -0,4597 |
|  | |  |  |  | |  |  | | |  | |  |  | |  | |  |  |
| SOC-R | | BA9M-R | -0,4908 | MGB-R | | BA9M-L | -0,4597 | | | CN-L | | BA47-R | -0,5537 | | MGB-L | | BA47-L | -0,4573 |
|  | |  |  |  | |  |  | | |  | |  |  | |  | |  |  |
| MGB-R | | BA9M-R | -0,5018 | MGB-R | | BA47-L | -0,4573 | | | MGB-L | | BA9M-R | -0,5018 | | CN-L | | BA45-L | -0,5174 |
|  | |  |  |  | |  |  | | |  | |  |  | |  | |  |  |
|  | |  |  | CN-R | | BA45-L | -0,5612 | | | SOC-L | | BA9M-R | 0,4908 | |  | |  |  |
| Tinnitus | | | | | | | | | | | | | | | | | | |
| Right | | | | | | | | Left | | | | | | | | | | |
| RR | | | | RL | | | | | LR | | | | | LL | | | | |
|  |  |  |  |  |  |  |  | | |  |  |  |  | |  |  |  |  |
| ROI |  | ROI | r | ROI |  | ROI | r | | | ROI |  | ROI | r | | ROI |  | ROI | r |
| SOC-R | | BA45-R | 0,6519 | IC-R | | BA47-L | 0,5629 | | | IC-L | | BA9DL-R | -0,6206 | | MGB-L | | BA46-L | 0,6376 |
|  | |  |  |  | |  |  | | |  | |  |  | |  | |  |  |
| CN-R | | BA47-R | 0,7011 | MGB-R | | BA46-L | 0,6447 | | | CN-L | | BA45-R | 0,759 | | MGB-L | | BA45-L | 0,6289 |
|  | |  |  |  | |  |  | | |  | |  |  | |  | |  |  |
| SOC-R | | BA47-R | 0,5852 |  | |  |  | | | SOC-L | | BA9M-R | 0,5589 | | CN-L | | BA45-L | 0,7131 |
|  | |  |  |  | |  |  | | |  | |  |  | |  | |  |  |
| MGB-R | | BA47-R | 0,5927 |  | |  |  | | |  | |  |  | |  | |  |  |
|  | |  |  |  | |  |  | | |  | |  |  | |  | |  |  |
| CN-R | | BA9M-R | 0,6474 |  | |  |  | | |  | |  |  | |  | |  |  |
|  | |  |  |  | |  |  | | |  | |  |  | |  | |  |  |
| SOC-R | | BA9M-R | 0,706 |  | |  |  | | |  | |  |  | |  | |  |  |
| Positive connectivity – Negative connectivity | | | | | | | | | | | | | | | | | | |
