# Supplementary material for: Reduced sound-evoked and resting-state BOLD fMRI connectivity in tinnitus
Source: Neuroimage Clin. 2018 Aug 31;20:637–49. doi: 10.1016/j.nicl.2018.08.029 (PMC6128096; doi:10.1016/j.nicl.2018.08.029)
Supplement: Supplementary file 5 — Supplementary material [file mmc5.docx]

# MATERIALS AND METHODS

The ethics committee of Tübingen University (faculty of medicine) and University Clinic Tübingen (ethical approval-number 264-2016BO1) approved this study. Written consent was obtained from all participants at their first visit. All methods are used according the clinical standard Process and Methods Guide 2016

<http://www.hqontario.ca/portals/0/documents/evidence/quality-standards/qs-process-guide-1610-en.pdf>

Data from all participants included in the study were used for all methods included. The recruitment criteria were defined prior to the study. For all participants all method parts were performed within an average period of 3 weeks maximal. All standard conditions were the same for volunteers and tinnitus patients.

### Participants

### From fifty-eight participants thirty-four were included in the study (see including and exclusion criteria Supplementary Table 2). 17 participants had tinnitus (36 ± 12 (mean ± standard deviation (SD)) years; 11 male and 6 female; 13 right-handed and 4 left-handed). The remaining 17 participants did not have tinnitus (32 ± 13 (mean ± SD) years; 11 male and 6 female; 15 right-handed and 2 left-handed). Participants had no known neurological disorders. The study participants were in two groups with a maximum of low-grade sensorineural hearing loss (hearing threshold not more than 40 dB per single frequency in the pure tone audiogram (PTA)).

Recruitment was performed through consecutive trials.

### Tinnitus questionnaire

The Goebel-Hiller-Score (G-H-S) tinnitus questionnaire with several questionnaire sub-scores in addition to the total tinnitus score was used to assess different aspects concerning tinnitus severity, laterality, emotional distress, cognitive distress, self-experienced intrusiveness, and auditory perceptual difficulty scores ([Hiller et al., 1994](#_ENREF_29)). All volunteers with tinnitus were asked to answer 52 statements in the questionnaire for different conditions that have impact on their lives, and they were asked to answer if this statement is true, partially true or not true. The different questionnaire scores were collected and every sub-score calculated according to the answers of the volunteers with tinnitus. The analyzed scores were then correlated with tinnitus loudness levels of all tinnitus participants to find out which aspects are related to tinnitus intensity measured during audiological evaluation.

In order to assess the presence of hyperacusis, a Hyperacusis Questionnaire ([Fischer, 2013](#_ENREF_17)) was administered to all participants.

Audiological evaluation

Ear examination was carried out by the study ENT physicians in the outpatient Ear, Nose and Throat (ENT) Department of the University Clinic Tübingen. Using ear cones and Zeiss ear microscopes, the ear canal was cleaned from wax and the ear canal and the tympanic membrane were inspected to exclude external canal anomaly or tympanic membrane pathologies.

Tympanometry and acoustic reflex measurements were performed in the Audiology Unit of the ENT Department with a Madsen-Zodiac 901 (GN Otometrics, Münster, Germany) using stimuli of 0.5, 1, 2, and 4 kHz for 80 to 100 dB sound pressure level (SPL) to ensure normal middle ear transmission.

Hearing thresholds were determined by pure tone audiometry (PTA) with the AT 900 Audiometer (Auritec, Medizindiagnostische Systeme GmbH, Hamburg, Germany). The audiometric testing was done in one-octave steps from 0.125 to 10 kHz. Test frequencies were presented to the single ear for descending sound pressure levels down to the participants hearing threshold. For the speech audiometry, the clinical standard test (‘Freiburger’) was used.

For the volunteers with tinnitus, sound of different frequencies and intensities of were presented to the individual ears to identify tone pitch and loudness closest to the perceived tinnitus. The tinnitus masking suppression volume, the sound level at which the tinnitus is masked or suppressed by a sine wave, was integrated to the figure of the pure tone audiogram.

The auditory evoked brainstem response (ABR) testing was done by using the system GSI Audera (Grason-Stadler, Eden Prairie, USA) device, with Telephonics TDH 39p headphones (Telephonics, Farmingdale, USA). Measurements were performed with two-channel recording using four electrodes (Neuroline 720, Ambu, Bad Nauheim, Germany) at predetermined positions according to the International Electrode System 1020 standard system after cleaning the volunteer’s skin with abrasive paste (Nuprep Skin Prep Gel, Weaver and Company, Aurora, USA) and attaching the electrodes with electrolytic paste and adhesive tape. The predetermined positions were active electrodes at the left mastoid (M1) and at the right mastoid (M2), a ground electrode at the forehead near the eyebrows, and a reference electrode centered close to the hairline as described in the manual of the above-mentioned device. The electrodes were connected to the pre-amplifier. The ABR was recorded ipsi-laterally in response to broadband acoustic click stimuli (0.1-ms duration) presented with 25 to 75 dB SPL in 10 dB steps. The click was presented at repetition rate of 11.1 Hz with 2000 repetitions. ABR signals were bandpass filtered between 150 Hz and 3000 Hz and recorded for 10 ms. Electrode impedance was maintained at less than 5 kΩ and not more than 2 kΩ impedance difference between two single electrodes.

Calculation of supra-threshold ABR wave fine structure

From the averaged ABR of a single ear and stimulus SPL, the absolute latencies and amplitudes of distinct components within the waveform of the ABR (positive deflections and subsequent negative deflections of the potential, “waves”) were extracted and attributed to their occurrences with respect to stimulus onset (defined as 0 ms). Wave amplitudes were determined between the leading positive and the trailing negative deflections (peaks) as the peak-to-peak amplitude differences (wave amplitudes) at predefined latencies of the leading positive peak. For time interval between 1-2 ms, 3-4 ms, 5-6 ms, and 6-7 ms waves I, III, V and VI were determined, respectively. In addition, the inter-wave latencies I-III, III-V and I-V were analyzed. The data for all the supra-threshold amplitudes, wave latencies and inter-wave latencies were analyzed for individual ears and the averaged for experimental groups (volunteer and tinnitus) for presentation and statistical analysis.

For salivary measurements

The saliva sample collection took place at three different day times – at 8:00 am, 4:00 pm and 11:00 p.m. The first saliva sample was collected in the ENT department at the first visit of the study at 4:00 p.m. (± 30 minutes). The two other samples were carried out by the participants at home (at the day before the fMRI took place). One sample at 8:00 a.m. and the other sample at about 11:00 p.m.

A Cotton Sallivete (No. 51.1534, Sarstedt, Nümbrecht, Germany) was used to collect approximately 1 ml for every sample collection. The participants were asked to keep a gentle chewing of the cotton swab for about one minute. Details of instruction about the sampling process was also explained. The Saliva samples were analyzed for cortisol in an extern laboratory (Limbach, Heidelberg, Germany).

Functional magnetic resonance imaging (fMRI)

MRI image acquisition was performed on a 3-Tesla scanner (Siemens, Magnetom 3-Tesla, Germany, Skyra) with a 20-channel head coil, in the Department of Interventional and Diagnostic Neuroradiology at the University Hospital of Tübingen, Germany. For the acoustic stimulation, we used special MRI-suitable over-ear headphones (CONFON HP-SC 03, MR Confon GmbH, Magdeburg, Germany). During scanning, five different auditory stimuli were generated using a stimulus presentation software (Neurobehavioral Systems software, [Neurobs,](https://www.neurobs.com) Berkeley, USA; Panasonic-SC-PMX5 Amplifier, Panasonic Marketing Europe GmbH, Hamburg, Germany).

The measurement experimental design of the task fMRI

The task-evoked functional images were obtained with a T2* weighted echo-planar sequence. The brain is moving due to the heart function, the brainstem even more than the cortex. To avoid artefacts or wrong/missing BOLD-signals we used cardiac gating. This is in general not common in case of upcoming signal variations by a non-constant TR time, which leads to superimpositions of the BOLD-effect and causes a disappearance of it. Therefore, a TR-correction procedure is necessary, which is described in the chapter “Task (evoked) functional magnetic resonance imaging analysis”. The image acquisition parameters were: repetition time (TR), 2000 to 3000 ms, depending on the cardiac pulse of the participant; flip angle (FA), 90 degrees; field of view (FOV), 290 mm; matrix size, 64x64; 10 coronal slices (as maximum to be within a heartbeat cycle and cover the auditory cortex/brainstem) with a slice thickness of 2.5 mm and 1.25 mm gap (distance between slices, 3.7 mm), echo time (TE), 35 ms.

Aiming at measuring the BOLD, in response to different acoustic stimuli, the presenter software version 16.1 (Neurobehavioral Systems software) was used to present four different acoustic stimuli with a specially written protocol. For the acoustic stimulation, we used special MRI-suitable over-ear headphones (CONFON HP-SC 03, MR Confon GmbH, Magdeburg, Germany). Those are equal to the common hearing-protection headphones, which were used during clinical MRI scans, but ensure that the participants hear the applied sound stimuli. The stimuli used are (1) high-frequency chirp (HF-chirp) with a range of 12000-20000 Hz, (2) low-frequency chirp (LF-chirp) with a range of 250-3000 Hz, (3) broadband chirp (BB-chirp) with a range of 300-25000 Hz, and (4) a rock music piece. All stimuli were used with cardiac gating. The pulse oximeter was applied to the participant’s finger to synchronize the heartbeats with the image acquisition, in order to improve the signal detection. The repetition time was 2 or 3 heartbeats dependent on the pulse rate of the participant. 15 blocks were used with alternating 8 off-blocks of rest and 7 on-blocks of active stimulus presentation, starting with the rest. For every block, 10 complete volumes were recorded with a resultant of 150 functional images in total.

The measurement experimental design of the resting state fMRI

The resting state functional images for the whole brain (including the brainstem – what is not common) were acquired over a 10 minutes acquisition time (TA) period of wakeful rest. Measurement parameters beside TR and number of slices were identical to task evoked. The measurements were not cardiac gated, the TR was 2 s, the number of slices per volume was 30. To get particular information about the resting state differences in lower brainstem (cochlear nucleus (CN), superior olivary complex (SOC), inferior colliculus (IC), we moved the FOV block (normally aligned to AC-PC line) of the excited slices toward lower brain regions, a technical detail until now not been performed for r-fcMRI studies in the auditory system. The participants were instructed to keep alert with eyes closed with no task to perform. Earplugs were used for all participants during the scan to reduce noise generated by the scanner. 300 volumes were acquired.

The T1-weighted three-dimensional structural images were obtained using MPRAGE sequence. The acquisition parameters were: TR, 2.3 s; TE, 2.1 ms; inversion time (TI), 900 ms; TA, 3.5 min; bandwidth, 200 Hz/pixel; FOV, 240 mm; slice thickness=0.94 mm and a 265 matrix.

Task (evoked) functional magnetic resonance imaging analysis

Compared to the often-used map-analysis (by bigger t-values in regions of stimuli-induced signal increases), we analyzed the fMRI data only by previously defined anatomical regions (Table 1). Additionally, this prevent the consideration of random occurrence of signal increases in the t-maps. The preprocessing steps were done for the images of all participants using SPM toolbox for MATLAB (version 8, Welcome Trust Center for Neuroimaging, UCL, London, GB) and the MATLAB programming system (version R2014a, MathWorks Inc., Natick, MA, USA). All the functional images were spatially realigned to the first image for head motion correction, then co-registration of the mean functional image with the structural image was done. After segmentation into white matter, grey matter and cerebrospinal fluid (CSF), all the realigned co-registered functional images were normalized to the Montreal Neurological Institute (MNI) template and the structural image. The normalized images were smoothed with the Gaussian Kernel of 5-mm full width at half maximum (FWHM). To detect correct single intensity for the variable TR, we used an own developed TR-correction script to make it possible to record even small BOLD-effects in brainstem regions. Therefore, we used the measured TR values for T1 estimation in each pixel, which could be used for a correction of single intensity in each voxel. All single intensities were calculated for a virtual TR of 2 s by an exponential fit ([Guimaraes et al., 1998](#_ENREF_25)). Afterwards the statistical model was specified in SPM.

In a first step, the general linear model (GLM) in SPM toolbox was used for single subject analysis. The specification of the general linear model design matrix, which defines our block experimental design and study hypothesis for every participant was done by first level specification of the smoothed normalized functional scans, followed by the estimation of the created general linear model. The result was acquired using contrast vectors to produce statistical parametric maps.

The group analysis of data from multiple subjects was also done to find out the difference in evoked central responsiveness between the volunteer and tinnitus group. The images of the first level specification were used in the second level analysis one-sample *t*-test of the SPM toolbox to average the volunteer and the tinnitus group separately, in order to investigate the local maxima of brain activity in response to different stimuli used. In a next step, the second level specification independent two-sample *t*-test of the SPM toolbox was used to perform the group analysis. Two different contrasts were used in the analysis. The first contrast is (volunteer > tinnitus) or (volunteer - tinnitus) that will represent the brain regions showing decreased or attenuated evoked response in the tinnitus group in comparison to the volunteer group, while the second contrast is (tinnitus > volunteer) or (tinnitus - volunteer) that will represent the brain regions showing increased evoked response in the tinnitus group in comparison to the volunteer group. ROIs where extracted out of these contrasts to represent significantly (*p<0.05*) increased or decreased brain areas in tinnitus group compared to volunteer group.

Resting state functional magnetic resonance imaging analysis

As like in the task-evoked analysis, the resting state signals where analyzed by previously defined anatomical regions (Table 1). The functional images were preprocessed and analyzed using the MATLAB programming system. The resting state functional images’ preprocessing and time course signal extraction were performed using the DPARSF_V2.3 MATLAB Toolbox ([Data Processing Assistant for Resting State fMRI](https://www.google.de/url?sa=t&rct=j&q=&esrc=s&source=web&cd=2&cad=rja&uact=8&ved=0ahUKEwitnoaz0-TOAhVRlxQKHQmED-UQFggjMAE&url=http%3A%2F%2Frfmri.org%2FDPARSF&usg=AFQjCNG2frBiE3J4pDl8_risIQfPSaKvkQ)) ([Chao-Gan and Yu-Feng, 2010](#_ENREF_8)). The parameters were set for slice timing, number of slices, slice-order and the reference slice to be used. All the functional images were spatially realigned to the first image for head motion correction, then co-registration of the mean functional image with the structural image was done. After segmentation into white matter, grey matter and CSF, all the realigned co-registered functional images were normalized to the MNI template and the structural image. The normalized images were smoothed with the Gaussian Kernel of 5 mm FWHM. Band-pass filtering was applied with 0.01 to 0.08 Hz. Effects of nuisance covariates as head motion parameters, global mean signal, white matter signal and cerebrospinal fluid signal were removed during the preprocessing. The time courses for all ROIs of all participants were successfully extracted in MATLAB files and the correlation matrix was calculated. The generated time course signals were used as an input to the MATLAB toolbox for analysis of functional brain connectivity (GraphVar) ([Kruschwitz et al., 2015](#_ENREF_38)). We tested against random networks, which is a non-parametric testing of the correlation strength (r) between a network with any user data against the correlation between the graph theoretical measures derived from the subject specific null-model network with random data (by using the same settings as the original network – i.e. threshold range, number of nodes).

After testing against random networks, the Pearson correlation matrix was generated that represent all the possible correlations in between time courses and can plot the significant correlations in a graph representing the significant functional connectivity pattern.
